# Supplementary material for: Single-cell data integration across weakly linked modalities
Source: PLoS Comput Biol. 2026 May 5;22(5):e1014231. doi: 10.1371/journal.pcbi.1014231 (PMC13160449; doi:10.1371/journal.pcbi.1014231)
Supplement: S1 File — (PDF) [file pcbi.1014231.s001.pdf]

Supporting information for "Single-cell data integration across weakly linked modalities"

Zhipeng Zhou, Yang Zhang, Zhiming Dai*

1 Methods for benchmarking

1. **Seurat** [1]: Through the identification of cell pairwise correspondences between single cells across datasets, termed "anchors", this method transforms datasets into a shared space. We built anchors of single-cell data based on the relationship between features (between different modalities) or features with the same name (between same modalities). The hyperparameters used in our experiment, and the preprocessing of different modal data are consistent with the "Integrative analysis in Seurat v5" vignette, which can be found in the GitHub repository `https://github.com/satijalab/seurat`. For cross-modality prediction, we employed the standard workflow using `FindTransferAnchors` to identify correspondences and `TransferData` to project reference features onto query cells.

2. **MARIO** [2]: This method takes into account both shared and specific features between modalities, while introducing quality control modules to generate joint embeddings that serve to cell matching. We referred to the documents provided in its GitHub repository `https://github.com/shuxiaoc/mario-py`. Under the guidance of its python tutorial file "MARIO matching and integration pipeline", we inputted the raw data file in csv format with the recommended hyperparameters given in this tutorial and completed the experiment. For cross-modality prediction, we utilized the generated cross-modality matching pairs to directly project reference features onto query cells.

3. **UniPort** [3]: This method combines coupled Variational AutoEncoder (coupled-VAE) and minibatch Unbalanced Optimal Transport (minibatch-UOT), utilizes highly variable general-purpose genes and dataset-specific genes for integration. We executed UniPort by following the procedures outlined in its GitHub repository `https://github.com/caokai1073/uniPort`. According to its "Diagonal integration with contrastive learning" tutorial document, we entered the raw data in h5ad format and took the hyperparameters of this tutorial as our experiment hyperparameters. For cross-modality prediction, we followed the "Impute genes for MERFISH"

tutorial and utilized the `Run` function with the parameter `out='predict'` to reconstruct the unmeasured features using the trained model.

4. **MaxFuse** [4]: This method is a cross-modal data integration method that, through iterative coembedding, data smoothing and cell matching, uses all information in each modality to obtain high-quality integration even when features are weakly linked. In the "Example of MaxFuse usage between RNA and Protein modality" tutorial presented in the GitHub repository `https://github.com/shuxiaoc/maxfuse?tab=readme-ov-file`, we learned how to use MaxFuse to integrate RNA and protein data. Then, using the raw data in csv format as input, we used MaxFuse to complete the integration and evaluation of other weakly and strongly linked modal datasets with the default hyper-parameter settings outlined in the above example. Similar to MARIO, we performed prediction by directly projecting reference features based on the identified cell matching pairs.

5. **scConfluence** [5]: This method combines uncoupled autoencoders, which reduce the dimensionality of the original data to a shared latent space and account for potential batch effects, together with regularized Inverse Optimal Transport (rIOT) [6]. We referred to "RNA_PROT_tutorial.ipynb" and "RNA_ATAC_pbmc_tutorial.ipynb" located in `https://github.com/cantinilab/scconfluence/tree/main`, in the weak linkage integration of "RNA + protein" and the strong linkage integration of "RNA + ATAC" respectively. After merging the data of multiple modalities and storing them in a unified h5mu format, we refer to the above two tutorials to preprocess the data of different modalities, set the hyperparameters, design and train the autoencoders, and finally get the latent embeddings for benchmarking. For cross-modality prediction, we referred to the "Gene imputation with scRNA and smFISH in mouse cortex" tutorial and employed the `impute_features` function to reconstruct the missing modality features based on the learned joint embeddings.

6. **CelLink** [7]: This method utilizes an iterative UOT framework specifically designed to address weak feature correlation and imbalanced cell populations in cross-modality integration. A functional advantage of CelLink is its ability to output batch-corrected feature matrices for downstream analysis. We referred to the documentation at its GitHub repository (`https://github.com/liu-bioinfo-lab/CelLink`) and tutorial website. While the benchmarking experiments were conducted using the recommended hyperparameter settings from the online tutorial, the raw data preprocessing followed the protocols detailed in the "Pre-process of the scRNA-seq, CODEX, and CITE-seq datasets" subsection of the main manuscript. This involved log-transformation, selection of the Highly Variable Genes (HVGs), and the exclusion of non-static features with standard deviations. For the scATAC-seq data, we converted the chromatin accessibility peaks into a gene activity matrix to align the feature space with gene expression data [8]. This was achieved by aggregating

peak counts located within the gene body and the 2kb upstream promoter region of the Transcription Start Site (TSS) for each gene, ensuring compatibility with CelLink's input requirements. For cross-modality prediction, we followed the "HPAP107 scRNA-seq and CODEX Integration" tutorial and retrieved the imputed feature matrices (stored in `feature_imputation_partition`) generated by the model, which utilize the learned transport maps to reconstruct the missing modality features.

7. **scMRDR** [9]: This method is a scalable generative framework for unpaired multi-omics integration that disentangles latent representations into modality-shared and modality-specific components using a regularized β-VAE architecture. It incorporates isometric regularization, adversarial training, and a masked reconstruction loss to effectively align modalities while handling missing features and preserving biological signals. We referred to the documentation and scripts provided in its GitHub repository `https://github.com/sjl-sjtu/scMRDR`. For the preprocessing of RNA, protein, and ATAC modalities, we followed the procedures outlined in the `experiments/BMMC_codes/data_clean.ipynb` file. The subsequent multi-modal integration and hyperparameter configurations were strictly consistent with the `Examples` section in the repository's `README.md` document. For cross-modality prediction, we referred to the `experiments/10x_paired_codes/integration_codes.py` script and utilized the `predict` function built into its `Integration` class to infer unmeasured features.

2 Details of datasets

This section provides a comprehensive overview of the datasets utilized in this study. To comprehensively validate MMIHCL, we employed eight datasets for technical benchmarking and two additional application-specific datasets for downstream biological discovery tasks. To facilitate a rigorous comparison, the eight benchmarking datasets were categorized into weakly and strongly linked scenarios based on the linkage ratio (ρ), as summarized in Table A. The two application-specific datasets were specifically introduced to evaluate disease classification and drug target discovery capabilities.

Building upon the quantitative metadata provided in Table A, detailed description of each dataset will be given next, including their biological significance, data acquisition platforms, and the specific sampling and preprocessing protocols (e.g., HVG selection and log-transformation) employed for our benchmarking experiments.

1. **CITE-seq PBMC** [10]: This dataset simultaneously assays 224 protein markers and whole transcriptome (20,729 RNAs) on Peripheral Blood Mononuclear Cells (PBMCs). The original dataset gives two levels of cell type annotation: 8 types of level 1, and 31 types of level 2, with the former chosen for discussion in our experiments and analysis. As in the previous

Table A: **Comprehensive statistical metadata and integration challenges of the eight benchmark datasets.**

Name	Modality 1	Modality 2	Linkage Number	Linkage Ratio (ρ)	Cell Type Number	Matching Scale	CTFSD	Linkage Type
CITE-seq PBMC [10]	10k cells × 20,729 RNAs	10k cells × 224 proteins	180	0.87%	8	1:1	0.1064	Weak
TEA-seq PBMC [11]	7k cells × 181352 RNAs	7k cells × 46 proteins	52	0.03%	8	1:1	0.0783	Weak
AB-seq BMC [12]	10k cells × 30,781 RNAs	10k cells × 97 proteins	96	0.31%	9	1:1	0.1184	Weak
CITE-seq BMC [10]	4k cells × 13,953 RNAs	4k cells × 134 proteins	114	0.82%	16	1:1	0.1121	Weak
CODEX tonsil [13, 14]	10k cells × 33,538 RNAs	30k cells × 46 proteins	53	0.16%	6	1:3	0.1289	Weak
CITE-seq & CyTOF PBMC [10, 15]	10k cells × 25 proteins	30k cells × 32 proteins	12	37.50%	7	1:3	0.0978	Strong
CyTOF human H1N1 & IFNG [16, 17]	10k cells × 41 proteins	10k cells × 40 proteins	39	95.12%	7	1:1	0.2180	Strong
10X-Multiome PBMC [18]	8k cells × 28,917 RNAs	8k cells × 13,205 ATACs	13,481	46.62%	10	1:1	0.1050	Strong

CTFSD refers to the Cell Type Frequency Standard Deviation, and is used to measure the unbalance of cell numbers among cell types in a dataset.

method MaxFuse [4], we respectively randomly sampled 10k of these cells from the original dataset for one-to-one matching.

2. **TEA-seq PBMC** [11]: This dataset is a paired human PBMCs dataset profiled by Transcription, Epitopes, and Accessibility sequencing (TEA-seq), which includes 46 protein markers. The cell type information was performed using R package Seurat [1] WNN-multi-modal clustering pipeline followed by manual annotation, and a total of 8 populations were identified. We sampled the RNA (18,352 features) and protein data of its 7k cells to conduct one-to-one matching.

3. **AB-seq BMC** [12]: This dataset is of cell-matched human Bone Marrow mononuclear Cells (BMCs) with an antibody panel of size 97 and the whole transcriptome (30,781 RNAs). The 20 cell types of original annotation were manually binned into 9 cell types in our experiments and analysis. We also performed a one-to-one matching of sampled 10k cells on it like CITE-seq PBMC.

4. **CITE-seq BMC** [10]: This dataset includes paired healthy BMCs profiled from different sites and donors with 13,953 gene expression features and 134 surface proteins. The 42 cell types of original annotation were manually binned into 16 cell types used for benchmarking. We conducted one-to-one matching-based integration on a subset of it containing 4k cells.

5. **CODEX tonsil** [13, 14]: This dataset consists of two parts: (1) CODEX tonsil images with 46 markers and (2) scRNA-seq tonsil disociated cells

(33,538 RNAs), whose cells are unpaired. The cell type annotation was also performed using R package Seurat [1] clustering pipeline like TEA-seq PBMC [11], and 6 common populations were used. We sampled 10k RNA and 30k protein cell data from them to look for one-to-three matching.

6. **CITE-seq & CyTOF PBMC** [10, 15]: This is a PBMC dataset measured in two healthy people using the CITE-seq and CyTOF respectively. These two techniques can measure 25 and 32 partially overlapping protein markers. We directly used the annotation information of 7 populations given by the original dataset. We sampled 10k and 30k cells from them respectively, and also got a one-to-three matching.

7. **CyTOF human H1N1 & IFNG** [16, 17]: We performed one-to-one matching of two CyTOF datasets from studies in which (1) human whole blood cells isolated from individuals challenged with H1N1 virus including 41 protein markers, (2) human whole blood cells stimulated with IFNG including 40 protein markers. Similarly, the original cell annotation information made up of 7 populations was used directly, and the sampling number in both datasets was 10k.

8. **10x-Multiome** [18]: This dataset simultaneously assays whole transcriptome (28,917 RNAs) and 13,205 regions of open chromatin on human PBMCs, of which we took 2,000 HVGs. After removing the rare cells (HPSC and Plasma), we binned the remaining 17 cell types of original annotation into 10 cell types. Then we performed a one-to-one matching of sampled 8k cells on it.

Beyond the benchmarking datasets described above, the following two datasets containing distinct biological conditions (batches) were utilized to assess MMIHCL's performance in disease classification and drug target discovery:

1. **HPAP** [19]: This dataset is derived from the Human Pancreas Analysis Program (HPAP) and consists of unpaired single-cell multi-omics data (scRNA-seq and protein/CyTOF) profiling pancreatic islets. Specifically, we selected samples from a Type 1 Diabetes (T1D) donor (ID: HPAP-023) and a healthy control donor (ID: HPAP-107) to construct the disease-state comparison. To construct the experimental dataset, we utilized all 278 available cells for the RNA modality of the T1D donor (HPAP-023). For the remaining groups, we performed uniform random downsampling, selecting 474 cells for the RNA modality of the control donor (HPAP-107) and 1,000 cells for the protein modality of both donors. Given the heterogeneity between donors, the raw data exhibited partially disjoint feature spaces and cell populations. To ensure a consistent integration benchmark, we intersected both the RNA and protein features as well as the cell type sets shared between the two donors. The resulting dataset, containing common cell types (including Alpha, Delta-PP, and Ductal), serves as a rigorous ground truth for evaluating MMIHCL's ability to distinguish pathological states.

2. **Kang18 PBMC** [20]: This dataset comprises single-cell RNA-seq data of PBMCs derived from eight lupus patients. The cells were divided into two conditions: a control group and a group stimulated with interferon-beta (IFN-β) for 6 hours. To construct the experimental dataset, we first separated the cells by condition and identified the top 3,000 HVGs for each group independently. Subsequently, we performed uniform random downsampling to select 2,000 cells from each condition, resulting in a balanced raw dataset. We utilized the condition labels (control vs. simulated) and the known Interferon-Stimulated Genes (ISGs) to validate MMIHCL's effectiveness in identifying drug targets.

3 Data preprocessing

For raw data of each modality, we uniformly preprocess it including normalization, logarithmization, and scaling. In addition, for modalities with higher feature dimensions, including single-cell RNA sequence (scRNA-seq) and single-cell Assay for Transpose-Accessible Chromatin sequence (scATAC-seq), we retain its HVGs to improve operational efficiency. These operations are implemented by the SCANPY [21] python library using default parameters. For more information on the preprocessing of different raw data, please refer to the public implementation of MMIHCL we published.

4 Experimental hyperparameter settings

All models were optimized using the PyTorch [22] library. We used the Adam [23] optimizer with a learning rate of 0.001, and the number of epochs for fitting model is selected from $\{500, 1000, 1500\}$, the default value is 1000. The dimension hyperparameters p'_x, p'_y, r_x, r_y, r and r^* used when using PCA and/or CCA are selected from $\{20, 25, 30\}$. The default value for p'_x and p'_y is 30, the default value for r_x, r_y, r and r^* is 20. The initial k value used to generate the adjacent cell graph is selected from $\{10, 15, 20\}$ (default 15). In the hypergraph embedding learning module, K is selected from $\{32, 48, 64\}$ (default 32), L is selected from $\{1, 2, 3\}$ (default 2), τ is selected from $\{0.1, 0.2, 0.5\}$ (default 0.1), λ is selected from $\{0.01, 0.02, 0.05\}$ (default 0.001). In the cell matching module, p is selected from $\{1, 2, 3\}$, α is selected from $\{0.5, 0.6, 0.7, 0.8\}$. Finally, the number of rounds of iterative optimization T is selected from $\{1, 2, 3\}$. More dataset-specific hyperparameter settings can be viewed in the public implementation of MMIHCL.

5 Details of evaluation metrics

Let $\mathbf{X}^* \in \mathbb{R}^{n_x \times r^*}$ and $\mathbf{Y}^* \in \mathbb{R}^{n_y \times r^*}$ denote the final joint embeddings for the two modalities, and $\mathbf{Z}^* \in \mathbb{R}^{(n_x+n_y) \times r^*}$ denote the vertically concatenated joint embedding matrix of $\mathbf{X}^*$ and $\mathbf{Y}^*$. To ensure reproducibility, we provide the

rigorous mathematical definitions for the seven quantitative metrics used in the evaluation framework as follows:

1. **ACCuracy (ACC)**: This metric measures the percentage of correctly matched pairs between modalities. Given the final predicted matching pairs $\mathbf{M}^*$, the ground truth cell type labels $\{l_i\}$, ACC is defined as:

$$\text{ACC} = \frac{|\{(i,j) \in \mathbf{M}^* \mid l_i = l_j\}|}{|\mathbf{M}^*|}. \tag{1}$$

2. **Fraction of Samples Closer Than True Matching (FOSCTTM)**: This metric quantifies the single-cell alignment error in the joint embedding space. For each cell i in modality $\mathbf{X}$ with a true paired cell j in modality $\mathbf{Y}$, the sample-wise error is calculated as the fraction of samples in $\mathbf{Y}$ that are closer to i than j:

$$\text{FOSCTTM} = \frac{1}{n_x + n_y} \left(\sum_{i=1}^{n_x} \frac{n_y^{(i)}}{n_y} + \sum_{j=1}^{n_y} \frac{n_x^{(j)}}{n_x} \right), \tag{2}$$

where $n_y^{(i)} = |\{k \mid d(x_i, y_k) < d(x_i, y_j)\}|$ represents the number of samples in modality $\mathbf{Y}$ that are closer to cell i (from modality $\mathbf{X}$) than j. The term $n_x^{(j)}$ is defined analogously for cell j in modality $\mathbf{Y}$ (counting closer samples in $\mathbf{X}$). Since a lower FOSCTTM indicates better matching performance, we utilize $1 - \text{FOSCTTM}$ in the calculation of the overall score ($\text{S}_{\text{overall}}$), to ensure consistency with the "higher-is-better" evaluation logic.

3. **Normalized Mutual Information (NMI)**: This metric evaluates the agreement between the clustering results of Z^* and the ground truth cell type labels. It is defined as:

$$\text{NMI} = \frac{2 \cdot I(C; L)}{H(C) + H(L)}, \tag{3}$$

where C denotes the cluster assignments of Z^*, L denotes the true cell labels, $I(\cdot)$ is the mutual information function, and $H(\cdot)$ is the entropy function. NMI ranges from [0, 1].

4. **Adjusted Rand Index (ARI)**: This metric evaluates the similarity between the predicted clusters of Z^* and true cell labels L, adjusted for chance. Given the raw Rand Index (RI), it is defined as:

$$\text{ARI} = \frac{\text{RI} - E[\text{RI}]}{\max(\text{RI}) - E[\text{RI}]}. \tag{4}$$

The theoretical range of ARI is [-1, 1], where negative values indicate that this predicted result is worse than random assignment. For the overall score ($\text{S}_{\text{overall}}$) calculation, we clip negative values to 0 (i.e., $\max(0, \text{ARI}) \in [0, 1]$).

5. **Average Silhouette Width for cell type labels ($\mathbf{ASW_{label}}$)**: This metric evaluates the separation of cell types in Z^*. We calculate the scaled average of the silhouette widths (s_i) across all N cells:

$$\text{ASW}_{\text{label}} = \frac{1}{2}\left(1 + \frac{1}{N}\sum_{i=1}^{N}\frac{b_i - a_i}{\max(a_i, b_i)}\right), \tag{5}$$

where a_i is the mean intra-cluster distance, and b_i is the mean nearest-cluster distance for cell i. $\text{ASW}_{\text{label}}$ is bounded within the range [0, 1].

6. **Graph Connectivity (GC)**: This metric evaluates the connectivity of cells with the same cell type within the k-Nearest Neighbor (kNN) graph of Z^*. It is defined as:

$$\text{GC} = \frac{1}{|T|}\sum_{c \in T}\frac{|\text{LCC}_c|}{N_c}, \tag{6}$$

where T is the set of cell types, LCC_c denotes the size of the largest connected component in the subgraph restricted to cells of type c, and N_c is the total number of cells of type c. GC ranges from [0, 1].

7. **Average Silhouette Width for batches ($\mathbf{ASW_{batch}}$)**: This metric evaluates the mixing of batches in Z^*. We calculate the silhouette widths based on batches and apply an absolute transformation to reward mixing:

$$\text{ASW}_{\text{batch}} = 1 - \left|\frac{1}{N}\sum_{i=1}^{N}\frac{b_i - a_i}{\max(a_i, b_i)}\right|, \tag{7}$$

where a_i is the mean intra-batch distance, and b_i is the mean nearest-batch distance for cell i. $\text{ASW}_{\text{batch}}$ is bounded within the range [0, 1].

References

[1] Stuart T, Butler A, Hoffman P, Hafemeister C, Papalexi E, Mauck WM, et al. Comprehensive integration of single-cell data. cell. 2019;177(7):1888-902.

[2] Zhu B, Chen S, Bai Y, Chen H, Liao G, Mukherjee N, et al. Robust single-cell matching and multimodal analysis using shared and distinct features. Nature Methods. 2023;20(2):304-15.

[3] Cao K, Gong Q, Hong Y, Wan L. A unified computational framework for single-cell data integration with optimal transport. Nature Communications. 2022;13(1):7419.

[4] Chen S, Zhu B, Huang S, Hickey JW, Lin KZ, Snyder M, et al. Integration of spatial and single-cell data across modalities with weakly linked features. Nature Biotechnology. 2024;42(7):1096-106.

[5] Samaran J, Peyré G, Cantini L. scConfluence: single-cell diagonal integration with regularized Inverse Optimal Transport on weakly connected features. Nature Communications. 2024;15(1):7762.

[6] Ma S, Sun H, Ye X, Zha H, Zhou H. Learning cost functions for optimal transport. arXiv preprint arXiv:200209650. 2020.

[7] Luo X, Huang Y, Tao Y, Feng F, Hopkirk A, Bate TSR, et al. CelLink: integrating single-cell multi-omics data with weak feature linkage and imbalanced cell populations. bioRxiv. 2024. Available from: `https://www.biorxiv.org/content/early/2024/11/22/2024.11.08.622745`. arXiv:https://www.biorxiv.org/content/early/2024/11/22/2024.11.08.622745.full.pdf. doi:10.1101/2024.11.08.622745.

[8] Stuart T, Srivastava A, Madad S, Lareau CA, Satija R. Single-cell chromatin state analysis with Signac. Nature methods. 2021;18(11):1333-41.

[9] Sun J, Liang C, Wei R, Zheng P, Bai L, Ouyang W, et al. scmrdr: A scalable and flexible framework for unpaired single-cell multi-omics data integration. arXiv preprint arXiv:251024987. 2025.

[10] Hao Y, Hao S, Andersen-Nissen E, Mauck WM, Zheng S, Butler A, et al. Integrated analysis of multimodal single-cell data. Cell. 2021;184(13):3573-87.

[11] Swanson E, Lord C, Reading J, Heubeck AT, Genge PC, Thomson Z, et al. Simultaneous trimodal single-cell measurement of transcripts, epitopes, and chromatin accessibility using TEA-seq. Elife. 2021;10:e63632.

[12] Triana S, Vonficht D, Jopp-Saile L, Raffel S, Lutz R, Leonce D, et al. Single-cell proteo-genomic reference maps of the hematopoietic system enable the purification and massive profiling of precisely defined cell states. Nature immunology. 2021;22(12):1577-89.

[13] Kennedy-Darling J, Bhate SS, Hickey JW, Black S, Barlow GL, Vazquez G, et al. Highly multiplexed tissue imaging using repeated oligonucleotide exchange reaction. European Journal of Immunology. 2021;51(5):1262-77.

[14] King HW, Wells KL, Shipony Z, Kathiria AS, Wagar LE, Lareau C, et al. Integrated single-cell transcriptomics and epigenomics reveals strong germinal center–associated etiology of autoimmune risk loci. Science immunology. 2021;6(64):eabh3768.

[15] Levine JH, Simonds EF, Bendall SC, Davis KL, El-ad DA, Tadmor MD, et al. Data-driven phenotypic dissection of AML reveals progenitor-like cells that correlate with prognosis. Cell. 2015;162(1):184-97.

[16] Rahil Z, Leylek R, Schürch CM, Chen H, Bjornson-Hooper Z, Christensen SR, et al. Landscape of coordinated immune responses to H1N1 challenge in humans. The Journal of clinical investigation. 2020;130(11):5800-16.

[17] Bjornson-Hooper ZB, Fragiadakis GK, Spitzer MH, Chen H, Madhireddy D, Hu K, et al. A comprehensive atlas of immunological differences between humans, mice, and non-human primates. Frontiers in immunology. 2022;13:867015.

[18] 10x Genomics. 10x Genomics Datasets. 10x Genomics; 2022. Available from: `https://www.10xgenomics.com/resources/datasets`.

[19] Fasolino M, Schwartz GW, Patil AR, Mongia A, Golson ML, Wang YJ, et al. Single-cell multi-omics analysis of human pancreatic islets reveals novel cellular states in type 1 diabetes. Nature metabolism. 2022;4(2):284-99.

[20] Kang HM, Subramaniam M, Targ S, Nguyen M, Maliskova L, McCarthy E, et al. Multiplexed droplet single-cell RNA-sequencing using natural genetic variation. Nature biotechnology. 2018;36(1):89-94.

[21] Wolf FA, Angerer P, Theis FJ. SCANPY: large-scale single-cell gene expression data analysis. Genome biology. 2018;19:1-5.

[22] Paszke A, Gross S, Massa F, Lerer A, Bradbury J, Chanan G, et al. Pytorch: An imperative style, high-performance deep learning library. Advances in neural information processing systems. 2019;32.

[23] Kingma DP. Adam: A method for stochastic optimization. arXiv preprint arXiv:14126980. 2014.
