# Supplementary figures and images for: Single-cell data integration across weakly linked modalities
Source: PLoS Comput Biol. 2026 May 5;22(5):e1014231. doi: 10.1371/journal.pcbi.1014231 (PMC13160449; doi:10.1371/journal.pcbi.1014231)

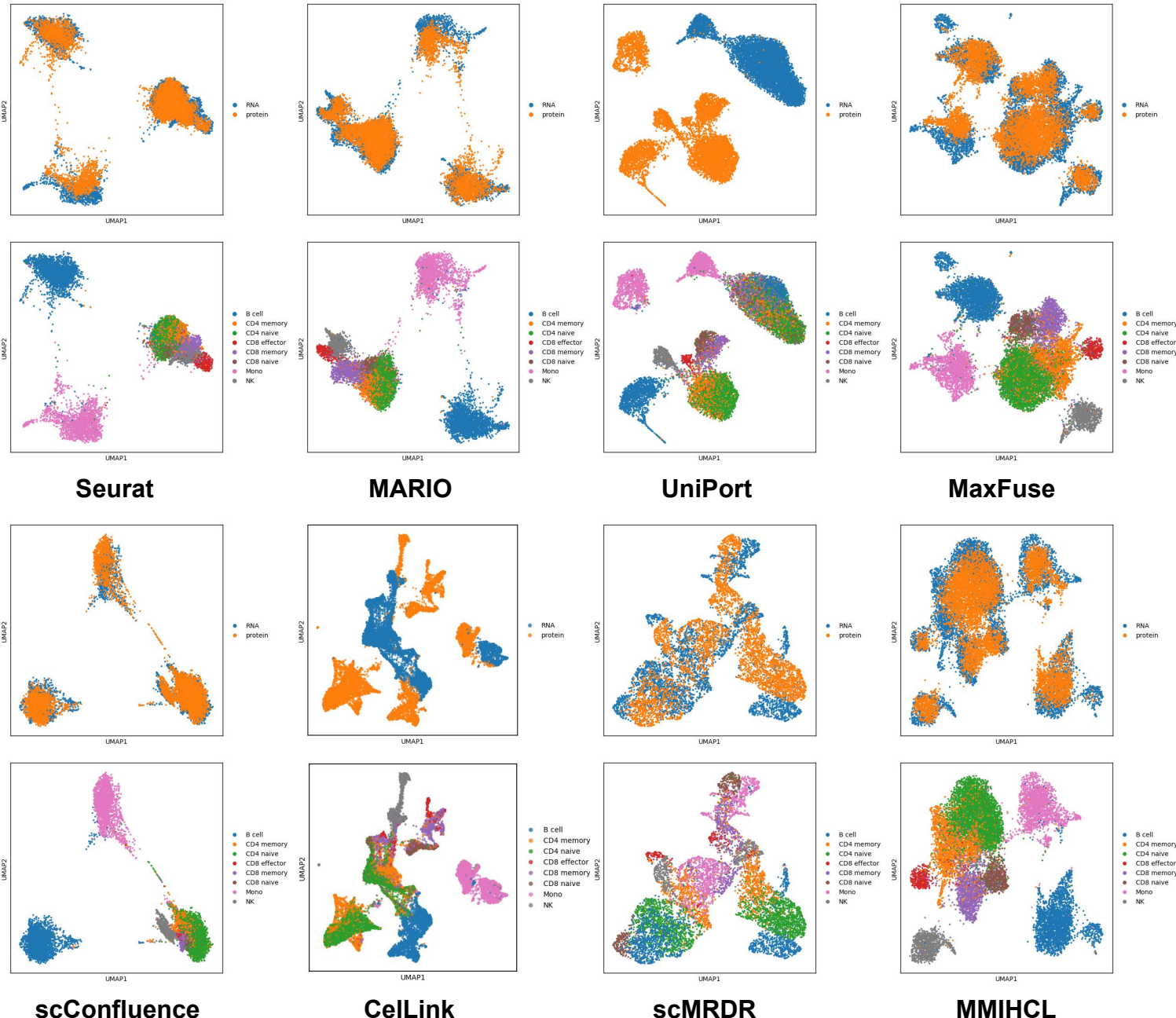

Supplement: S1 Fig — The first and third row subgraphs are colored by data modality, and the second and fourth row subgraphs are colored by cell type. Other UMAP graphs in supporting information are also arranged in this way. (PDF) [file pcbi.1014231.s003.pdf]

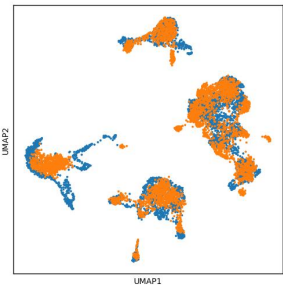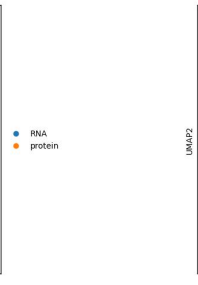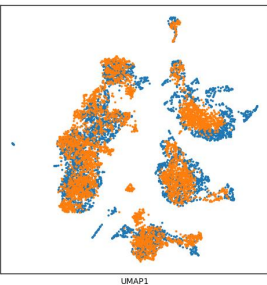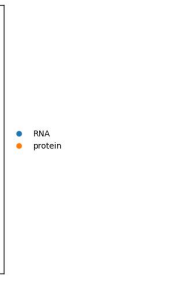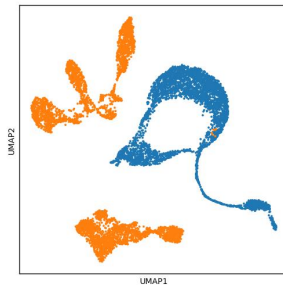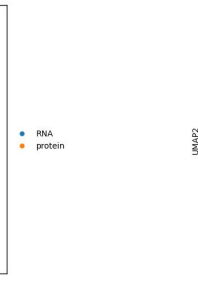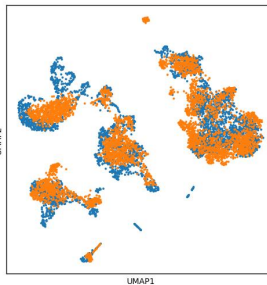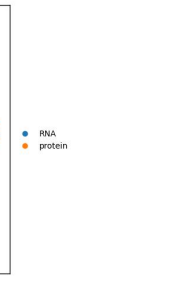

**Seurat**

**MARIO**

**UniPort**

**MaxFuse**

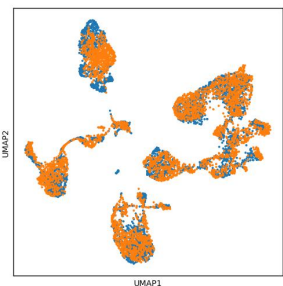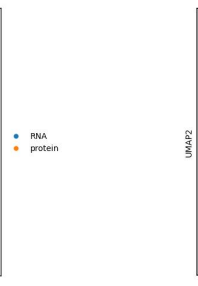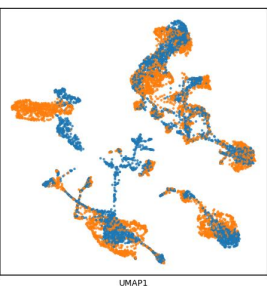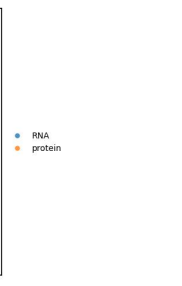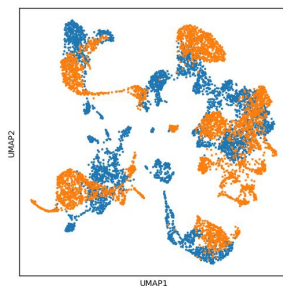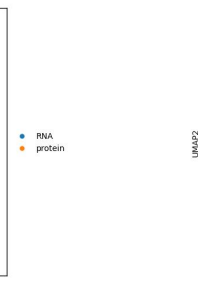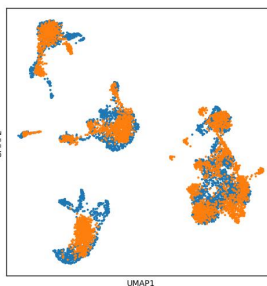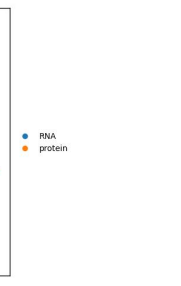

**scConfluence**

**CellLink**

**scMRDR**

**MMIHCL**

Supplement: S3 Fig — (PDF) [file pcbi.1014231.s005.pdf]

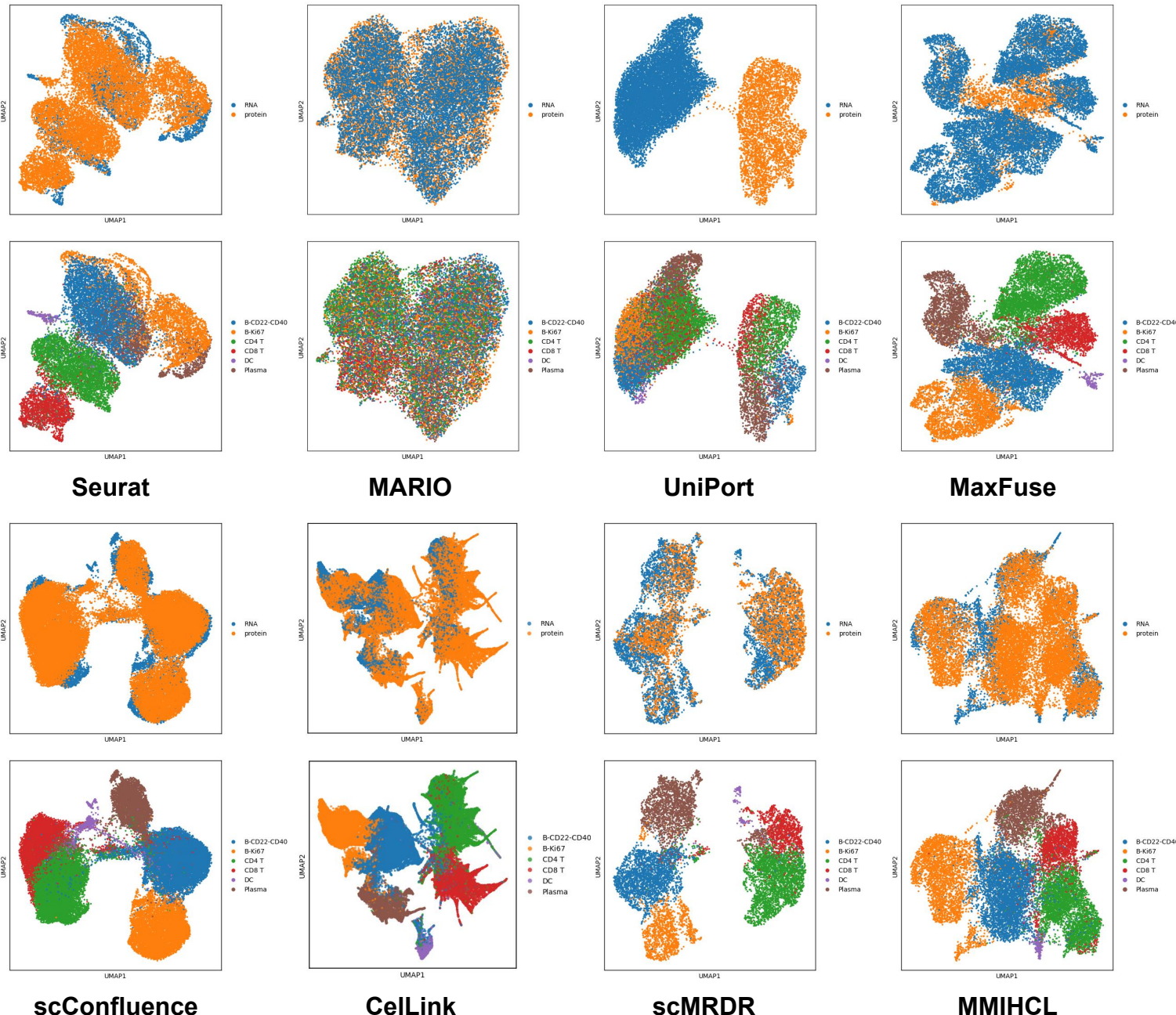

Supplement: S4 Fig — (PDF) [file pcbi.1014231.s006.pdf]

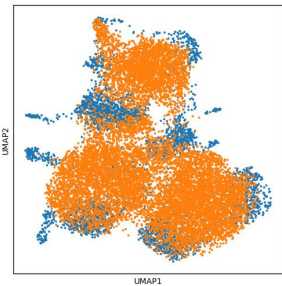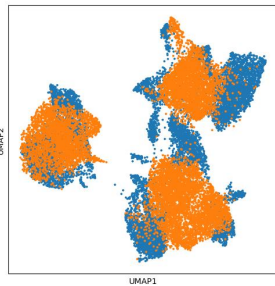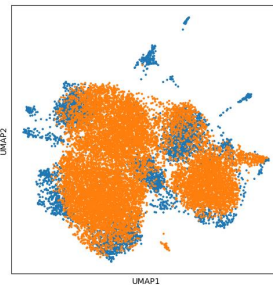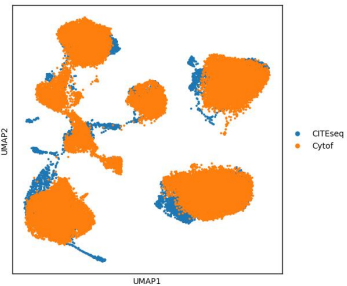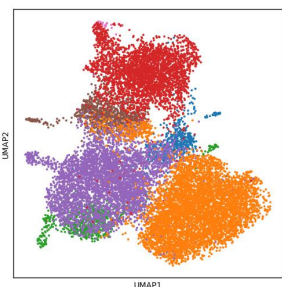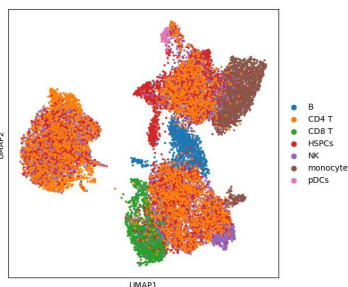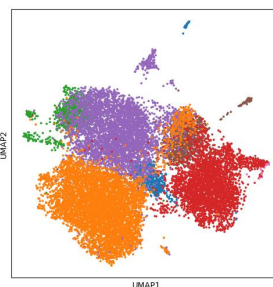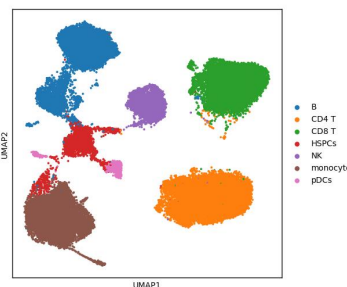

**Seurat**

**MARIO**

**MaxFuse**

**scConfluence**

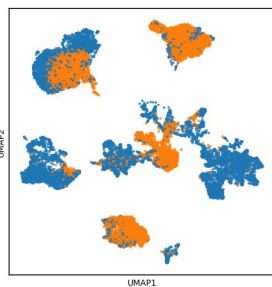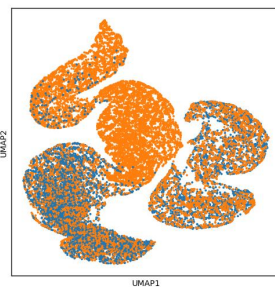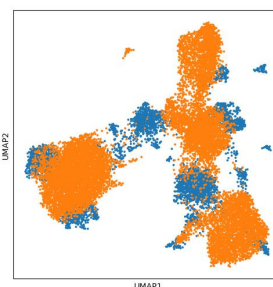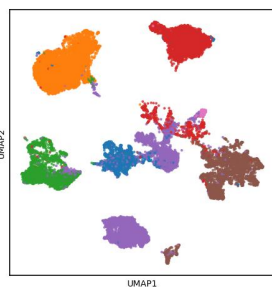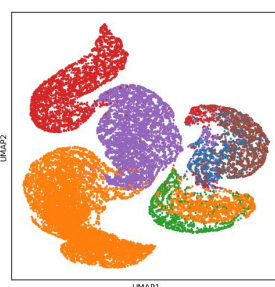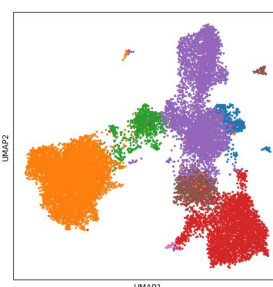

**CellLink**

**scMRDR**

**MMIHCL**

Supplement: S5 Fig — (PDF) [file pcbi.1014231.s007.pdf]

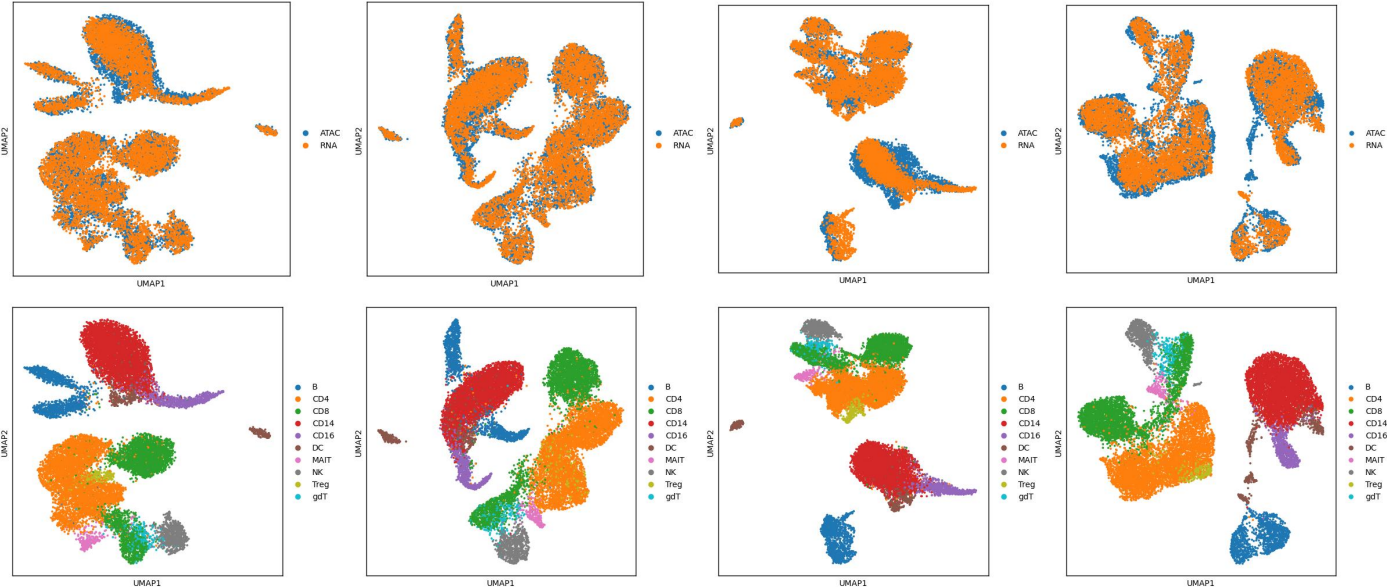

**Seurat**

**MARIO**

**MaxFuse**

**scConfluence**

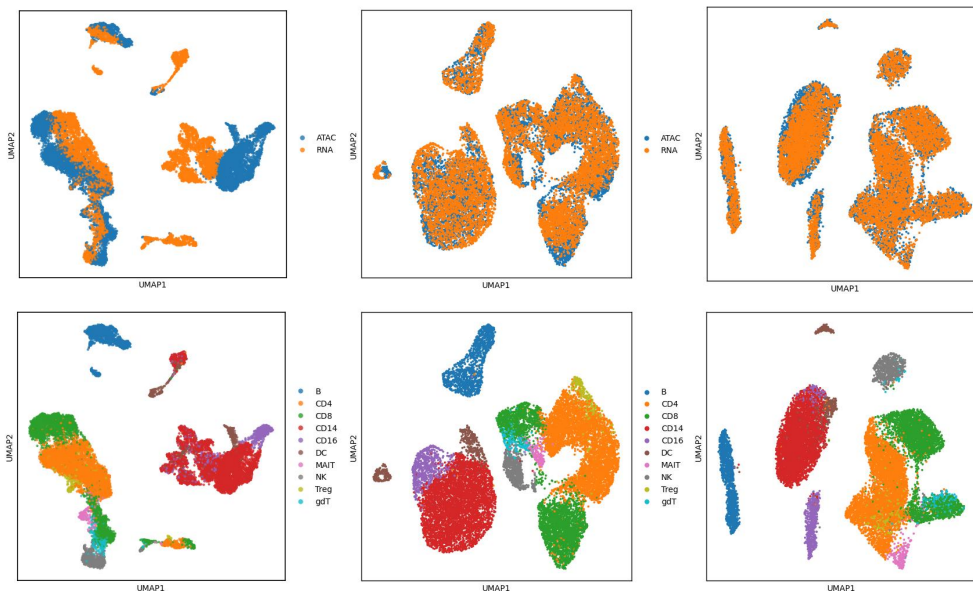

**CellLink**

**scMRDR**

**MMIHCL**

Supplement: S6 Fig — (PDF) [file pcbi.1014231.s008.pdf]

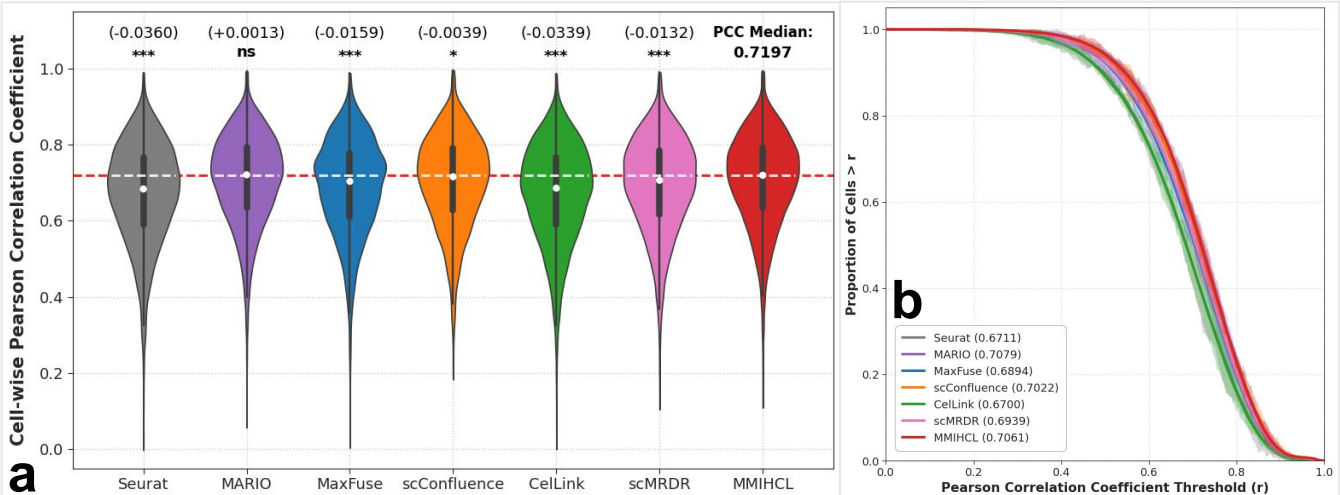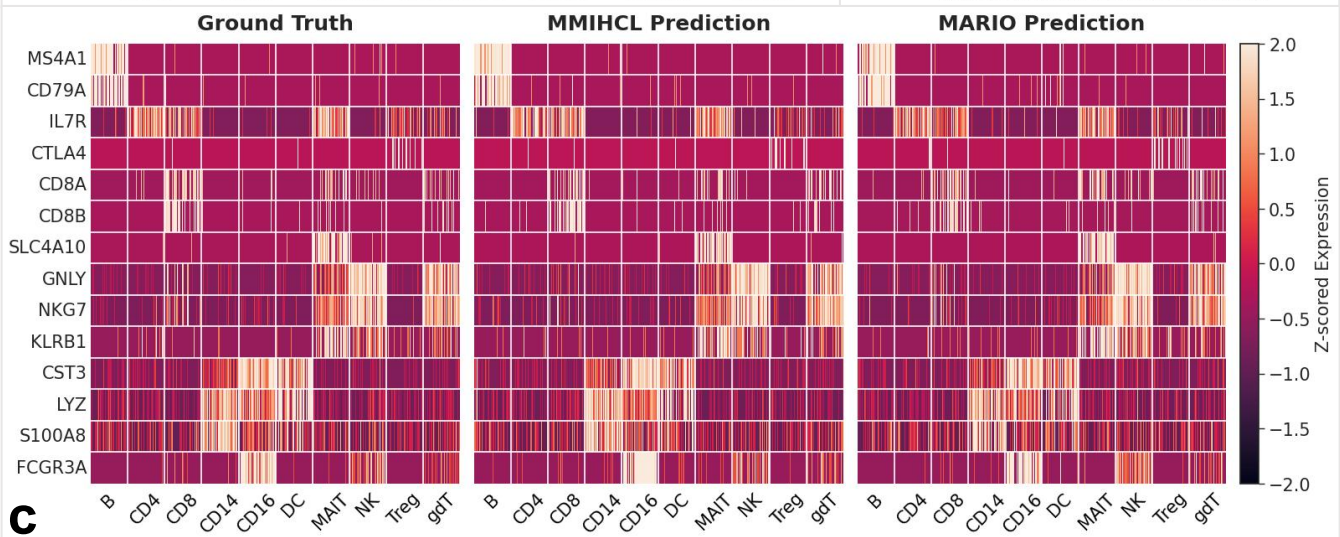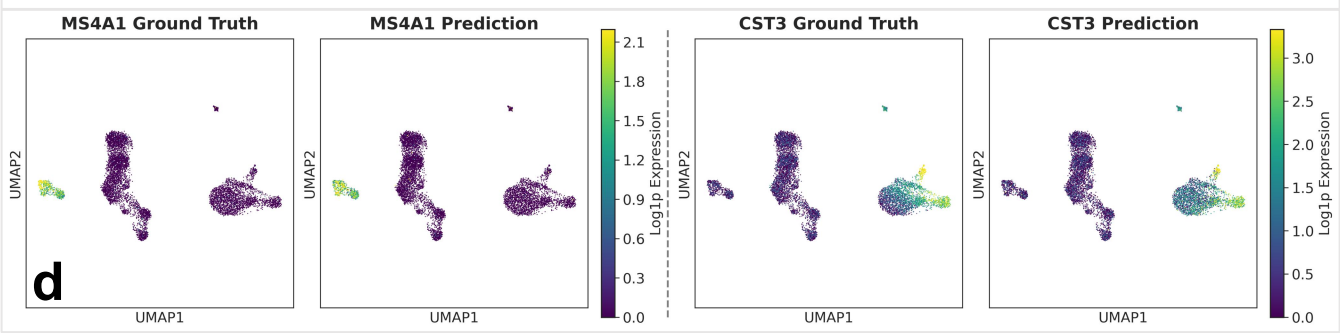

Supplement: S7 Fig — (a) Violin plots displaying the distribution of cell-wise Pearson Correlation Coefficients (PCCs) between ground truth and predicted gene expression profiles. The white dot represents the median PCC, and the thick bar indicates the interquartile range. The numbers above the violins indicate the difference in median PCC relative to MMIHCL. Statistical significance was determined using two-sided Wilcoxon signed-rank tests (***: P < 0.001, **: P < 0.01, *: P < 0.05, ns: not significant). (b) CDF curves illustrating the proportion of cells (y-axis) surpassing specific PCC thresholds (x-axis). The translucent shading surrounding each curve represents the standard deviation, and the values in parentheses within the legend denote the AUC for each method. (c) Side-by-side heatmaps comparing the z-scored expression of representative marker genes (e.g., MS4A1, CST3, CD8A) across annotated cell types for ground truth, MMIHCL prediction, and MARIO prediction. Rows represent gene markers, and columns represent individual cells sorted by cell type. (d) UMAP visualizations of ground truth versus predicted expression for two lineage-specific gene markers: MS4A1 (B cells) and CST3 (Monocytes). (PDF) [file pcbi.1014231.s009.pdf]

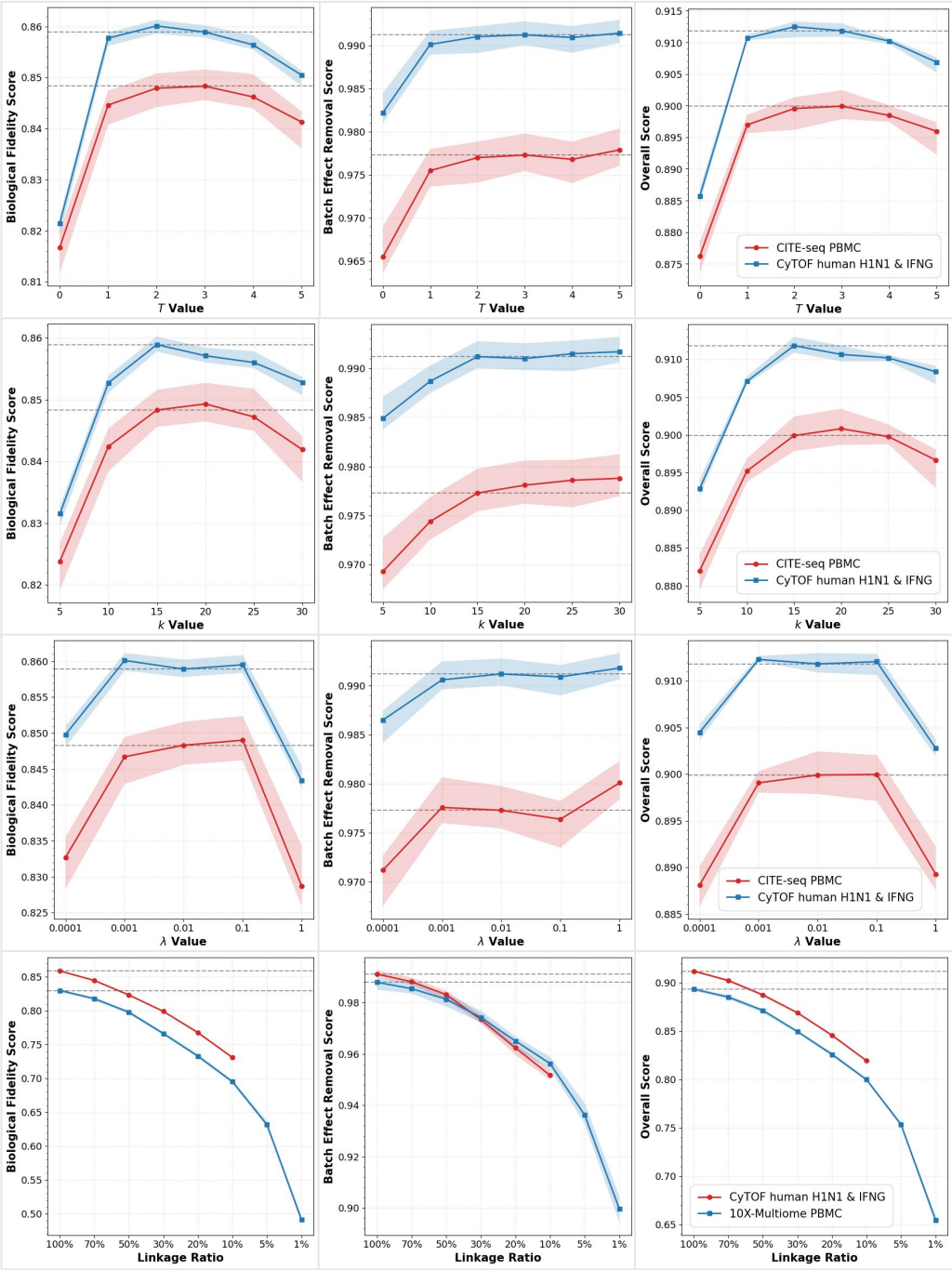

Supplement: S9 Fig — Rows and columns correspond to four hyperparameters (T, k, λ, plink) and three evaluation metrics (Sbio, Sbatch, Soverall), respectively. Solid lines represent the mean of five independent runs, while shaded areas indicate the minimum-maximum range. (PDF) [file pcbi.1014231.s011.pdf]
