# Supplementary material for: Single-cell data integration across weakly linked modalities
Source: PLoS Comput Biol. 2026 May 5;22(5):e1014231. doi: 10.1371/journal.pcbi.1014231 (PMC13160449; doi:10.1371/journal.pcbi.1014231)
Supplement: S2 Fig — (PDF) [file pcbi.1014231.s004.pdf]

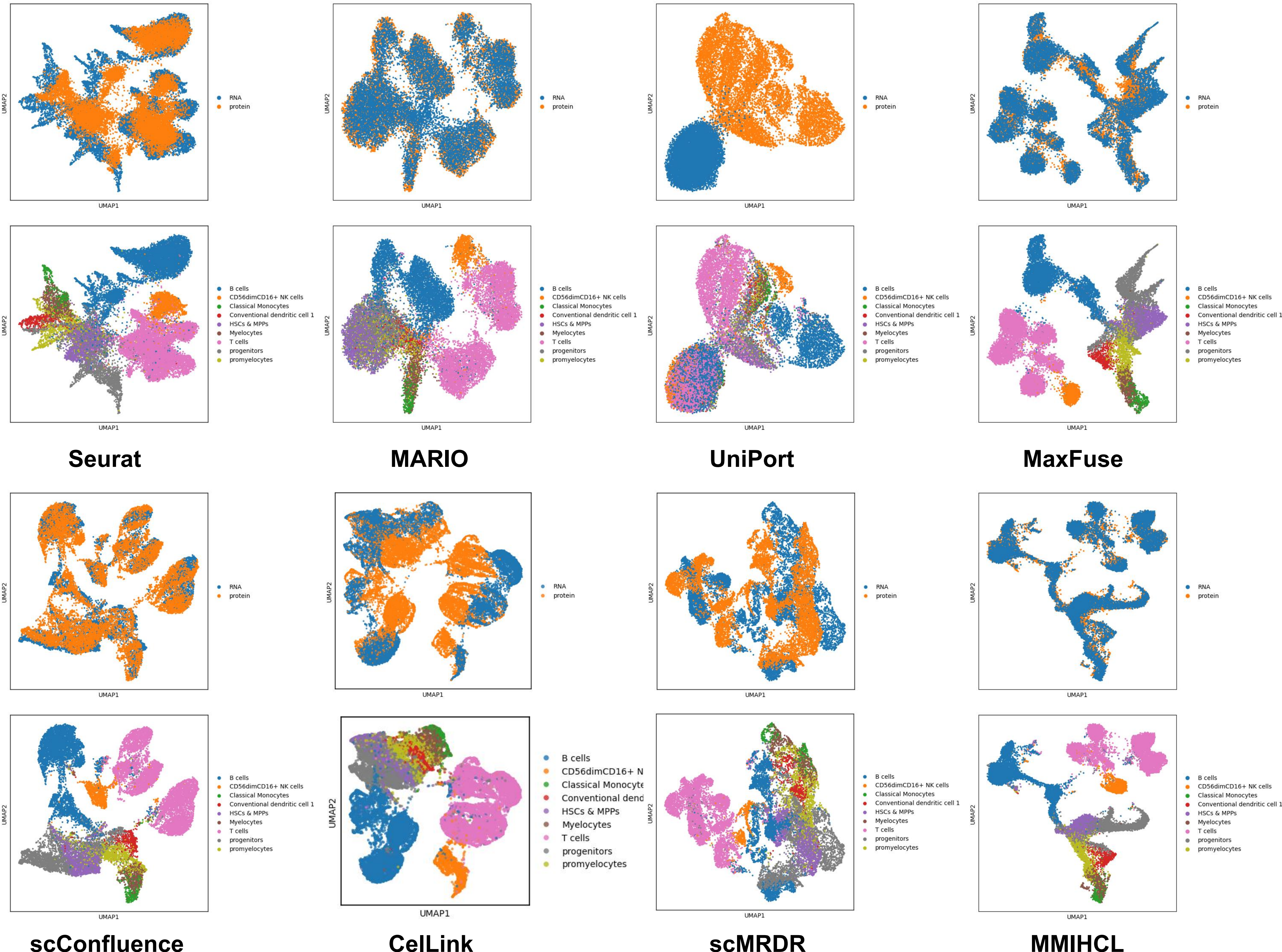
UMAP1
UMAP2
RNA
protein
B cells
CD56dimCD16+ NK cells
Classical Monocytes
Conventional dendritic cell 1
HSCs & MPPs
Myelocytes
T cells
progenitors
promyelocytes
Seurat
MARIO
UniPort
MaxFuse
scConfluence
CellLink
scMRDR
MMIHCL
