# Supplementary material for: Single-cell data integration across weakly linked modalities
Source: PLoS Comput Biol. 2026 May 5;22(5):e1014231. doi: 10.1371/journal.pcbi.1014231 (PMC13160449; doi:10.1371/journal.pcbi.1014231)
Supplement: S8 Fig — To analyze the neighbor distribution, the adaptive graphs were constructed using the primary modality of each dataset: CyTOF for CITE-seq & CyTOF PBMC, H1N1 for CyTOF human H1N1 & IFNG, and RNA for the remaining datasets. For each dataset, the bar plot illustrates the proportion of different cell types, highlighting the prevalence of class imbalance. The corresponding box plots display the distribution of adaptive k values (derived from an initial k = 30) assigned by the akNN module. The Spearman’s Rank correlation (RS) and P-value annotated in each panel indicate a consistent and significant positive relationship between cluster size and neighbor counts across all datasets. (PDF) [file pcbi.1014231.s010.pdf]

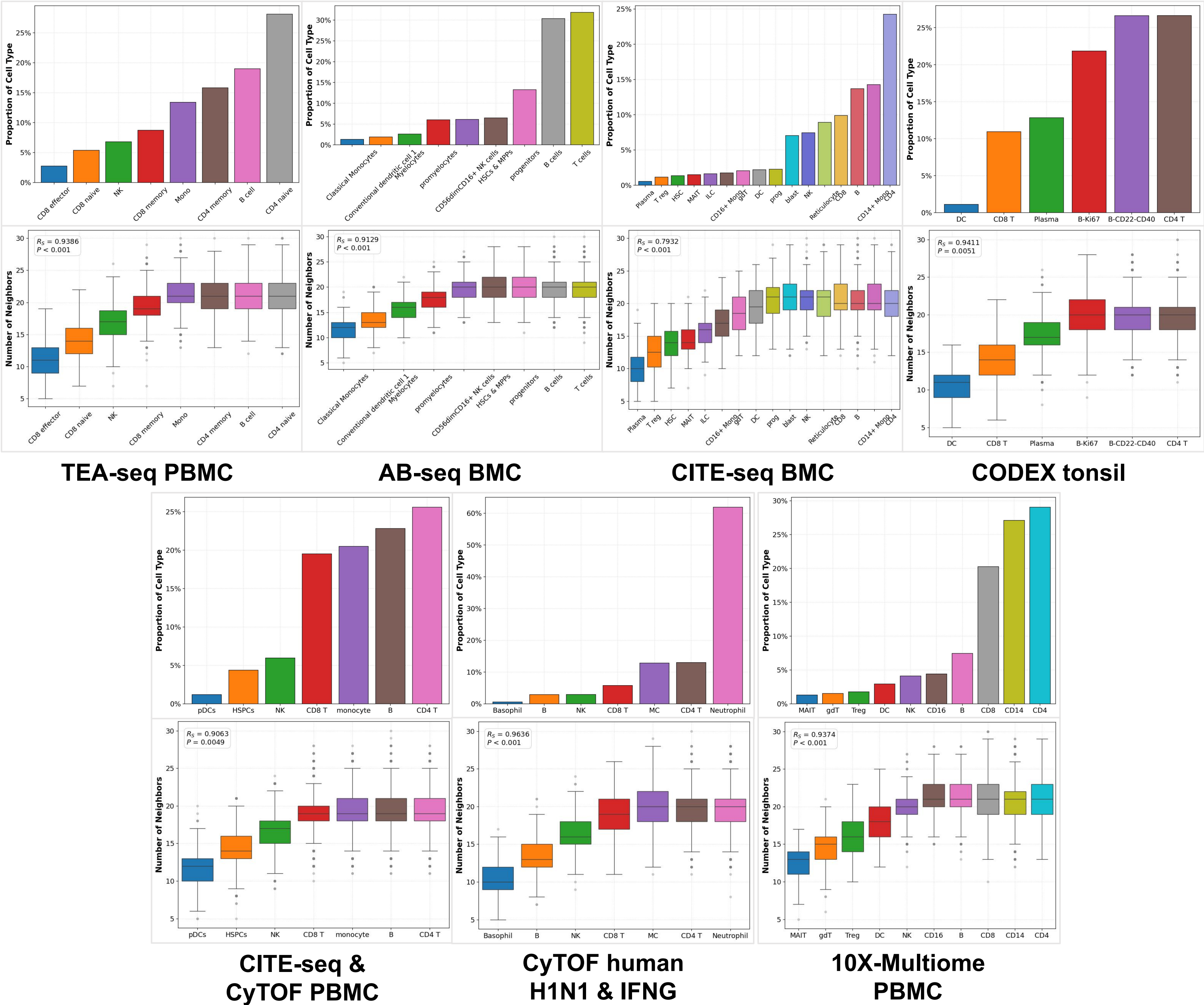

Proportion of Cell Type
CD8 effector
CD8 naive
NK
CD8 memory
Mono
CD4 memory
B cell
CD4 naive
Number of Neighbors
R_S = 0.9386
P < 0.001
TEA-seq PBMC
Classical Monocytes
Conventional dendritic cell 1
Myelocytes
promyelocytes
CD56dimCD16+ NK cells
HSCs & MPPs
progenitors
B cells
T cells
R_S = 0.9129
P < 0.001
AB-seq BMC
Plasma
T reg
HSC
MAIT
ILC
CD16+ Mono
gdT
DC
prog
blast
NK
Reticulocyte
CD8
B
CD14+ Mono
CD4
R_S = 0.7932
P < 0.001
CITE-seq BMC
DC
CD8 T
Plasma
B-Ki67
B-CD22-CD40
CD4 T
R_S = 0.9411
P = 0.0051
CODEX tonsil
pDCs
HSPCs
NK
CD8 T
monocyte
B
CD4 T
R_S = 0.9063
P = 0.0049
CITE-seq & CyTOF PBMC
Basophil
B
NK
CD8 T
MC
CD4 T
Neutrophil
R_S = 0.9636
P < 0.001
CyTOF human H1N1 & IFNG
MAIT
gdT
Treg
DC
NK
CD16
B
CD8
CD14
CD4
R_S = 0.9374
P < 0.001
10X-Multiome PBMC
