# Supplementary material for: Single-cell data integration across weakly linked modalities
Source: PLoS Comput Biol. 2026 May 5;22(5):e1014231. doi: 10.1371/journal.pcbi.1014231 (PMC13160449; doi:10.1371/journal.pcbi.1014231)
Supplement: Attachment — Submitted filename: Response to Reviewers.pdf [file pcbi.1014231.s013.pdf]

Response to Editor and Reviewers for PCOMPBIOL-D-25-01825

Zhipeng Zhou, Yang Zhang, and Zhiming Dai

1 General Response

Dear Editor and Reviewers,

We thank the Editor and Reviewers for the thoughtful and detailed feedback provided on our manuscript (PCOMPBIOL-D-25-01825). We have carefully considered each comment and have revised the manuscript to strengthen the theoretical framework and empirical validation of MMIHCL.

In addition to the scientific revisions, we have addressed the Journal Requirements as follows:

- The **working link** for CyTOF PBMC dataset has been updated in the submission system (the previous invalid link was our mistake), to ensure full data availability.
- The **Financial Disclosure** statement has been updated in the submission system (including the required "to ZD" initials and the funder role disclaimer) to comply with the journal's specific requirements.

The following sections provide our point-by-point responses to each of the comments. Please note that unless otherwise specified, all Page and Line numbers cited in this Response Letter refer to the **Revised Manuscript with Track Changes**.

Sincerely,
Zhipeng Zhou, Yang Zhang, and Zhiming Dai

2 Response to Additional Editor Comments

Comment 1:

The pipeline in Fig. 1 should be revised to improve readability, with less text and a clearer presentation of the workflow.

Response 1:

We sincerely appreciate the Editor's constructive suggestion regarding the visual presentation of our pipeline. We apologize for the initial version of **Fig. 1**, which indeed contained redundant descriptive text and an overly narrative caption, that may have hindered a quick

understanding of the MMIHCL workflow. In accordance with the Editor's advice, we have shifted the figure's presentation style from "text-heavy description" to "notational logic" and have refined the corresponding caption accordingly. These changes provide a much clearer and more professional overview of the MMIHCL framework.

Changes in Manuscript 1:

Firstly, let us explain the structural and visual improvements made to **Fig. 1** (Page [6]). As illustrated in the side-by-side comparison between the original pipeline (**Fig. R1**) and the revised version (**Fig. R2**), we have performed a comprehensive overhaul of the schematic representation of MMIHCL:

- **Transition from Narrative to Notational Logic:** We replaced verbose descriptive phrases (e.g., input X, Calculate Correlation) with standardized mathematical notations (e.g., $\mathbf{X} \in \mathbb{R}^{n_x \times p_x}$, $\mathbf{A_x} \in [0,1]^{n_x \times n_x}$). This ensures the figure is consistent with the rigorous mathematical definitions in the **Materials and methods** section.
- **Optimization of Iterative Refinement Workflow:** In subfigure (c), the previously linear layout has been redesigned into a circular workflow. By introducing curved arrows to connect the Hypergraph Learning $H(\cdot)$, M-to-M Matching, and CCA modules, we more intuitively demonstrate the cyclic nature of the refinement process ($t = 1, 2, \ldots, T$).
- **Refinement of Embedding Branches:** In subfigure (e), we simplified the "Message Passing" labels and the dual-branch architecture ($\bar{\mathbf{e}}$ and $\tilde{\mathbf{e}}$). This makes the contrastive learning mechanism and the subsequent fusion step more prominent.

As a result of these modifications, the text density in **Fig. 1** has been significantly reduced, meeting the Editor's requirement for improved readability and a clearer presentation of the workflow.

Secondly, we have overhauled the caption of to **Fig. 1** match this notation-based logic. The revised caption has been reconstructed to emphasize mathematical precision and clarity:

- **Shift to Notation-Based Description:** We replaced narrative sentences with formal mathematical symbols (e.g., $\mathbf{X}, \mathbf{Y}$), to ensure the caption precisely defines the variables visualized in the figure and matches the formalisms in the **Materials and methods** section.
- **Clarification of Iterative Refinement Workflow:** The revised text explicitly defines the iteration index (t), and the final convergence products ($\mathbf{X}^*, \mathbf{\Pi}^*$), providing a clearer logical mapping for the Stage 3 circular workflow.
- **Refinement of Module Details:** Descriptions of the akNN and hypergraph modules were also refined to highlight core mechanisms, such as the transition to the adaptive k_i parameter (consistent with Eq (4)), and the dual-branch contrastive learning structure.

To maintain the layout's aesthetic integrity and readability as requested, we have provided the fully rewritten caption in the "Track Changes" manuscript without the struck-through original text. A comprehensive side-by-side comparison is provided below for transparency.

- **Original:** (a) The input consists of two matrices of two modalities, some of whose features are linked. The two modal matrices are used to generate their own adjacency cell graphs through adaptive adjacent construction. (b) MMIHCL first uses linked feature embeddings learned by hypergraph embedding for initial many-to-many matching. (c) Then Canonical Correlation Analysis (CCA), hypergraph embedding learning and

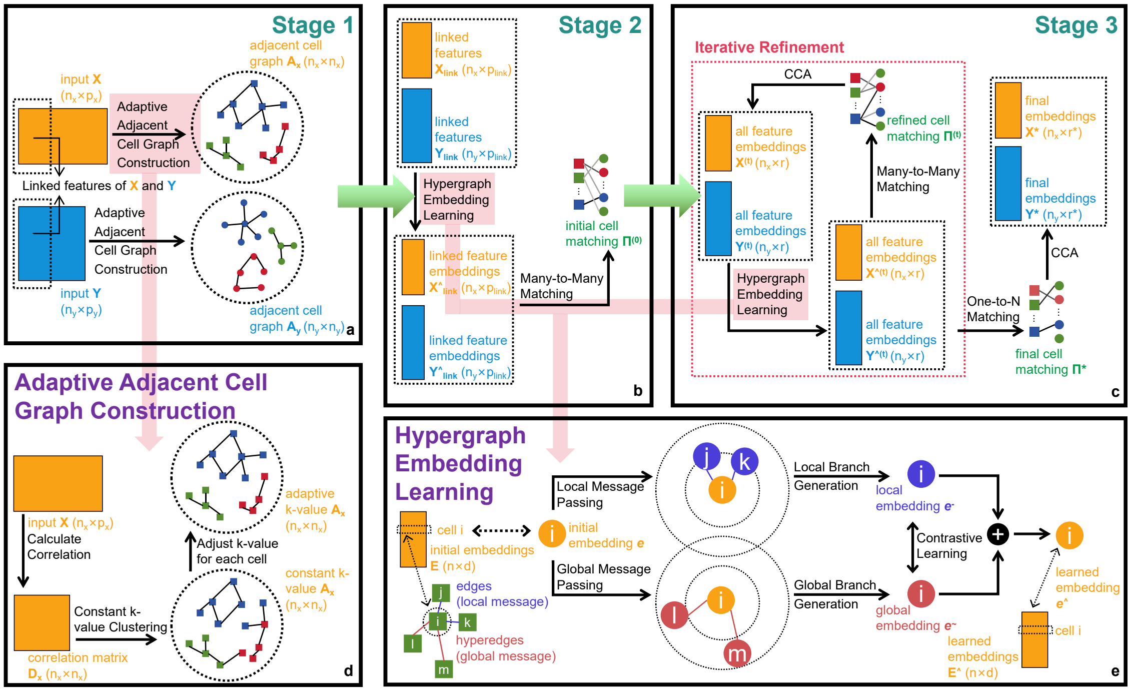

Figure R1: Original overview pipeline of MMIHCL.

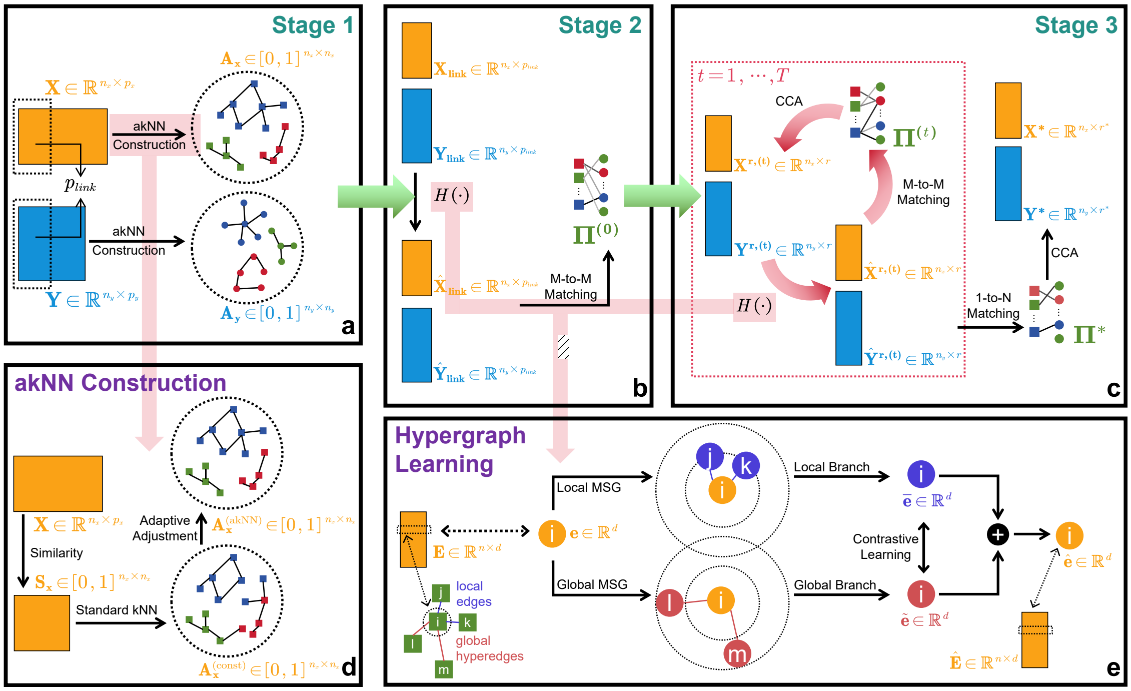

Figure R2: Revised overview pipeline of MMIHCL.

many-to-many matching are performed iteratively to generate the final matching and joint embeddings. (d) The details of adjacent cell graph construction. Use the all feature matrix to calculate the cell-wise feature correlation matrix between cells. Based on the

resulting matrix, the graph construction module first identifies the k-nearest neighbor of each cell, and then adaptively adjusts the k-value for each cell. (e) The details of hypergraph embedding learning. In the hypergraph embedding learning module, the two embedding branches of each cell are generated from local and global perspectives respectively. After contrastive learning, the embeddings from the two branches are summed to obtain the learned embedding for each cell.

- **Revised:** (a) The input modality matrices $\mathbf{X}$ and $\mathbf{Y}$ share p_{link} linked features. Weighted adjacency cell graphs $\mathbf{A_x}$ and $\mathbf{A_y}$ are constructed through the akNN module. (b) Linked features are embedded via the hypergraph operator $H(\cdot)$ to generate initial embeddings, followed by a Many-to-Many (M-to-M) matching process to obtain the initial matching $\mathbf{\Pi}^{(0)}$. (c) The workflow iteratively updates cell embeddings and matching for $t = 1, 2, \cdots, T$ iterations. Each loop involves $H(\cdot)$, Canonical Correlation Analysis (CCA), and M-to-M matching. The final outputs are the optimized joint embeddings $\mathbf{X}^*, \mathbf{Y}^*$ and the 1-to-N matching $\mathbf{\Pi}^*$. (d) Feature similarity $\mathbf{S_x}$ is calculated to transition from a constant k in standard kNN to a cell-specific adaptive k_i, yielding the final graph $\mathbf{A}_\mathbf{x}^{(\text{akNN})}$. (e) For a target cell i, local and global message-passing branches generate embeddings $\bar{\mathbf{e}}$ and $\tilde{\mathbf{e}}$, respectively. Contrastive learning is employed between the two branches to enhance representation robustness, followed by a fusion step to obtain the final learned embedding $\hat{\mathbf{e}}$.

Comment 2:

A rigorous and quantitative definition of "strong" versus "weak" linkage between modalities should be provided.

Response 2:

We thank the Editor for this insightful suggestion. We agree that a formal mathematical definition is essential for clarifying the conceptual framework of our study. Accordingly, we have established a rigorous quantitative categorization based on a newly defined linkage ratio ρ. We have selected 10% as a unified threshold to clearly demarcate the integration scenarios. This categorical boundary ensures a continuous and mutually exclusive classification across all evaluated datasets, thereby enhancing the robustness of our comparative analysis.

Changes in Manuscript 2:

In response to the Editor's request for a strict definition of linkage intensity, we have revised the manuscript to establish a unified and mutually exclusive quantitative categorization based on the linkage ratio ρ.

Firstly, in the **Results** section, we have added the mathematical definition of weak linkage to ensure clarity (Page [5], Line [136-137]).

- **Original:** We defined two datasets of modalities to be weakly linked if the number of their linked features is less than 10% of the number of all features in at least one modality.
- **Revised:** We categorize the linkage intensity between two modalities based on the linkage ratio ρ defined in Eq (1). Specifically, two datasets are defined as weakly linked if $\rho < 10\%$.

Secondly, in the **Results** section, we have updated the definition of strong linkage to

align with the unified 10% threshold, ensuring a continuous categorization (Page [10], Line [202-204]).

- **Original:** We defined two datasets of modalities to be strongly linked if the number of their linked features is more than 40% of the numbers of all features in both modalities, and then we also tested whether MMIHCL show competitive performance on strongly linked modalities.
- **Revised:** We also tested whether MMIHCL shows competitive performance on strongly linked modalities, which, according to the criteria in Eq (1), are defined as scenarios where $\rho \geq 10\%$.

Thirdly, in the **Materials and methods** section, we have formalized the criteria for both integration scenarios within the methodological framework. The summary of the new content is as follows (See Page [26], Line [539-545] for details):

- We have introduced a quantitative definition of the linkage ratio (ρ) to formally categorize the integration scenarios based on the intensity of shared features.
- Specifically, the linkage ratios for two modalities are defined as $\rho_x = p_{link}/p_x$ and $\rho_y = p_{link}/p_y$. Here, p_{link} signifies the number of linked features, while p_x and p_y denote the total feature counts of two modalities respectively.
- Based on these ratios, we categorized the scenarios into weak linkage ($\rho = \min(\rho_x, \rho_y) < 10\%$) and strong linkage ($\rho = \min(\rho_x, \rho_y) \geq 10\%$).

Comment 3:

Several typographical issues remain (e.g., inconsistent use of quotation marks in the Author Summary), in addition to those already noted by other Reviewers.

Response 3:

We sincerely apologize for the typographical issues. We have carefully addressed the typographical issues pointed out by the Editor and the Reviewers.

Changes in Manuscript 3:

- **Author Summary:** We have standardized the usage of quotation marks in the **Author Summary** (changing to consistent English double quotation marks "...").
- **Other Typographical Issues:** We have corrected all other typographical issues noted by the Reviewers throughout the manuscript (please refer to the response to the Reviewers for details).
- **Additional Check:** We have also performed an additional grammar, symbol, and layout check to ensure the manuscript meets the publication standards.

Comment 4:

The statement that the method "outperforms state-of-the-art methods" is overused and insufficiently informative. Instead, the authors should emphasize the key conceptual contributions, empirical findings, and distinctive features of MMIHCL. While reporting performance gains (e.g., 1–4%) is necessary, such marginal improvements alone do not constitute a mechanistic or methodological innovation.

Response 4:

We sincerely appreciate this critical and constructive observation. We fully accept the point that relying on broad claims of "outperforms state-of-the-art methods" is insufficient to convey the true scientific value of our work. We acknowledge that our original manuscript focused disproportionately on marginal performance gains in benchmark metrics, thereby obscuring the methodological innovations and practical utility of MMIHCL.

To address this, we have fundamentally restructured the manuscript to shift the narrative from a "ranking-oriented" perspective to a "utility-oriented" one. Moving beyond a primary focus on benchmark performance, we now position it as a robust engine for biological discovery. This transformation is realized through the following three key dimensions:

The first key dimension is **Narrative Shift: From Ranking to Utility**. We have revised the **Abstract**, **Author summary**, **Introduction**, and **Discussion** sections to minimize generic comparisons and instead highlight MMIHCL's mechanistic innovations, specifically its "Explicit Cell Matching" and "Dual-view Hypergraph Learning". We now frame these mechanisms not just as means to improve alignment scores, but as essential features that solve the fundamental challenge of "weak linkage" in multi-omics data. The revised narrative emphasizes that MMIHCL's value lies in its ability to facilitate downstream biological applications, transforming technical alignment into meaningful biological insights.

The second key dimension is **Introduction of Novel Empirical Findings**. To substantiate the method's practical capabilities, we have introduced two major application-specific modules that were absent in the original version:

- **Enabling Cross-Modality Feature Prediction (Fig. 6):** We demonstrated MMIHCL's unique capability to act as a "cross-omics translator". By leveraging its explicit cell matching mechanism, MMIHCL can accurately predict missing modalities (e.g., predicting protein abundance from RNA). This goes beyond simple data alignment, proving that the model learns a biologically meaningful mapping between disparate feature spaces.
- **Empowering Downstream Biological Discovery (Fig. 7):** We applied MMIHCL to real-world disease and perturbation datasets (HPAP and Kang18 PBMC). The results show that MMIHCL serves as a high-sensitivity signal filter. In disease stratification tasks, it successfully disentangles pathological states from technical noise without confounding cell type identities. In drug target discovery, it uncovers subtle but critical molecular signals (such as the precise differential expression patterns of canonical interferon-stimulated genes like *CXCL10*, *CXCL11*, and *CCL2*) that are often obscured by noise in other methods.

The third key dimension is **Methodological Rigor: From Single Metrics to Multi-Dimensional Evaluation**. Moving away from proving superiority via marginal metric improvements, we have established a comprehensive multi-dimensional evaluation framework.

- **Balanced Evaluation Framework:** We introduced a weighted overall score (S_{overall}) combined with radar charts and bio-vs-batch trade-off plots. This new evaluation strategy demonstrates that MMIHCL's distinct advantage lies in achieving an optimal balance between biological fidelity and batch effect removal, rather than simply maximizing one metric at the expense of another.
- **Computational Cost Robustness:** We provided a rigorous analysis of computational scalability, demonstrating MMIHCL's linear efficiency and memory stability in large-

scale scenarios where some competing methods fail due to resource exhaustion.

By highlighting these conceptual contributions and empirical findings, we believe the revised manuscript now presents a compelling scientific story that goes far beyond a standard benchmarking report.

Changes in Manuscript 4:

For the **Narrative Shift: From Ranking to Utility**, We have synchronized the narrative across the **Abstract**, **Author Summary**, **Introduction**, and **Discussion** sections:

- **Abstract & Author summary:** Rewrote the texts to explicitly feature the "Explicit Cell Matching" and "Dual-view Hypergraph Learning" mechanisms, replacing previous generic statements about outperforming state-of-the-art methods.
- **Introduction:** Reorganized the logical flow to frame "weak linkage" as a fundamental problem solved by our specific methodological innovations, rather than a leader-board challenge.
- **Discussion:** Updated the interpretation of results to align with the new findings, specifically discussing how the proposed mechanisms directly enable the downstream biological discoveries presented in the revised **Results** section.

For the **Introduction of Novel Empirical Findings**, we have added the relevant new subsections with visualizations:

- **MMIHCL enables accurate cross-modality feature prediction via explicit cell matching: Fig. 6** (Page [14]) quantitatively visualizes the prediction capability from RNA to protein modalities. Added analysis demonstrates MMIHCL's capability to map disparate feature spaces beyond simple alignment.
- **MMIHCL empowers robust disease classification and drug target discovery: Fig. 7** (Page [16]) comprises UMAP visualizations of disease stratification (HPAP dataset) and violin/volcano plots for drug response analysis (Kang18 PBMC dataset). Added analysis details the preservation of cell-type structures under pathological conditions and the recovery of specific Interferon-Stimulated Genes (ISGs) and other Differentially Expressed Genes (DEGs).

For the **Methodological Rigor: From Single Metrics to Multi-Dimensional Evaluation**, we have expanded the experimental content and updated the presentation format:

- **Benchmarking Exhibition:** Replaced single-metric boxplots with radar charts (revised **Fig. 2** (Page [8]) & **Fig. 4** (Page [11])) and bio-vs-batch 2D scatter plots to illustrate the trade-off between biological fidelity and batch correction.
- **Scalability Analysis:** Added **Fig. 9** (Page [23]) and a corresponding paragraph to rigorously evaluate runtime and memory usage, highlighting the linear scalability of MMIHCL compared to baseline methods.

Comment 5:

The authors suggest that MMIHCL can be applied to integrative studies such as disease classification and drug target discovery. To substantiate these claims, one or two real-world application examples should be included.

Response 5:

We sincerely appreciate this critical observation. Upon reflection, we recognize that in our original manuscript—particularly in the **Author Summary**—we suggested the potential of MMIHCL for downstream tasks like disease classification and drug target discovery without providing the necessary experimental validation. We fully agree that making such broad claims regarding biological applicability without direct empirical evidence is scientifically inadvisable and weakens the validity of the study.

To substantiate our claims with concrete evidence, we have significantly expanded the scope of our evaluation by incorporating two real-world, application-specific scenarios. These additions are designed not merely as supplementary examples, but as rigorous proofs-of-concept to demonstrate MMIHCL's utility in translational research:

- **Validating Disease Classification (HPAP Dataset):** We introduced a complex single-cell multi-omics dataset from the Human Pancreas Analysis Program (HPAP), specifically characterizing the heterogeneity between Type 1 Diabetes (T1D) donors and healthy controls. The challenge here is to distinguish pathological states in an unsupervised manner without confounding them with cell-type identity. Our new analysis demonstrates that MMIHCL successfully learns disease-associated latent representations. By generating high-quality joint embeddings, the model effectively segregates T1D samples from controls while strictly preserving the biological distinctness of islet cell subtypes, thereby proving its capability for precise, automated disease classification.
- **Validating Drug Target Discovery (Kang18 PBMC Dataset):** To test the model's sensitivity to subtle molecular perturbations, we employed the Kang18 dataset, which captures the immune response of PBMCs to interferon-beta (IFN-β) stimulation. In this scenario, the biological signal of the drug response is often masked by technical noise and the dominant signals of cell types. Acting as a robust signal filter, MMIHCL utilizes joint embeddings to construct a kNN graph that guides gene expression smoothing. This denoising enhances the signal-to-noise ratio, enabling the accurate recovery of obscured Interferon-Stimulated Genes (ISGs). Consequently, significant therapeutic targets were identified from the smoothed profile, validating MMIHCL's ability to extract meaningful biological signals from noisy data.

By moving from theoretical assertions to empirical validation, we believe the revised manuscript now solidly supports the claim that MMIHCL empowers robust downstream biological discovery.

Changes in Manuscript 5:

Firstly, we have introduced a comprehensive subsection titled **MMIHCL empowers robust disease classification and drug target discovery** in the **Results** section. This subsection details two key experiments:

- **Disease Classification Analysis (Page [17], Line [306-320]):** We detailed the application of MMIHCL on the HPAP dataset. We reported that MMIHCL achieves the clearest separation between T1D and control samples. Quantitatively, this is substantiated by achieving the highest Status NMI (0.4618) and ARI (0.5439) compared to SOTA baselines, confirming its superiority in distinguishing pathological states.
- **Drug Target Identification (Page [17-18], Line [321-338]):** We described the

recovery of drug response signals in the Kang18 dataset. We explicitly stated that MMIHCL effectively smooths biological noise to recover canonical ISGs. Specific marker genes, including *CXCL10*, *CXCL11*, and *CCL2*, were highlighted as being accurately recovered in the MMIHCL-enhanced expression profile.

Secondly, we have added a new multi-panel figure (**Fig. 7**, Page [16]) to visually support these claims:

- **Fig. 7a:** Displays the UMAP visualization of the integrated HPAP dataset, color-coded by disease status, showing a distinct separation between T1D (HPAP-023) and Control (HPAP-107) donors while maintaining cell type coherence in T1D donors.
- **Fig. 7b:** Presents a comparison of gene expression recovery for key ISGs (e.g., *CXCL10*) across different methods, visually demonstrating the superiority of MMIHCL's denoising capability.
- **Fig. 7c:** Shows a volcano plot derived from the MMIHCL-smoothed data, highlighting significantly the up-and-down regulated drug targets and confirming the statistical significance of the identified biological signals.

Thirdly, we have updated the **Experimental datasets** subsection in the **Materials and methods** section to briefly introduce these new resources. We explicitly stated (Page [33], Line [702-707]): "Beyond these benchmarking datasets, we incorporated two application-specific datasets, including HPAP and Kang18 PBMC, characterized by distinct biological conditions to validate MMIHCL in downstream discovery tasks. Unlike the standard benchmarking datasets, these two datasets contain condition-specific batch information, serving as the basis for disease classification and drug target discovery experiments."

Finally, we have significantly expanded **Details of datasets** in **S1 File** to provide technical specifications:

- **HPAP (Page [5], Line [151-167]):** We added details regarding the specific donors (T1D: HPAP-023 vs. Control: HPAP-107), the exact cell counts used for each modality, and the intersection strategy applied to features and cell types (including Alpha, Delta-PP, and Ductal) to ensure a rigorous comparison.
- **Kang18 PBMC (Page [5], Line [168-177]):** We clarified the experimental design involving IFN-β stimulation, detailing the preprocessing steps such as the independent identification of the top 3,000 Highly Variable Genes (HVGs) and the balanced downsampling to 2,000 cells per condition used to validate drug target discovery.

3 Response to Reviewer #1

Comment 1:

The authors define "weak linkage" as scenarios where linked features comprise less than 10% of the total features. While this is a practical working definition, it would be beneficial to explore the lower bound of this performance. I recommend conducting a sensitivity analysis where the number of available linked features is artificially downsampled (e.g., using only 1%, 5%, 10% of shared genes/proteins) on a strong-linkage dataset, which would powerfully demonstrate the robustness of the "weak linkage" definition.

Response 1:

We sincerely thank the Reviewer for this insightful suggestion. We agree that verifying the performance lower bound under "weak linkage" conditions is crucial for establishing the robustness of our definition. We acknowledge that our previous experimental design primarily measured model performance on standard weak and strong linkage datasets using their full set of linked features. This approach lacked a granular exploration of how performance degrades when only a fraction of linked features is available, leaving the stability boundary of the model unexplored.

To address this, we conducted a new sensitivity analysis using two representative strong linkage datasets: CyTOF human H1N1 & IFNG (protein & protein) and 10X-Multiome PBMC (RNA & ATAC). We artificially downsampled the linkage ratio from 100% down to extreme sparsity levels to simulate severe weak linkage scenarios. For the CyTOF human H1N1 & IFNG dataset, we set the lower bound at 10%. This is because 10% corresponds to only ≈ 4 linked features; further reduction to 1% would result in no linked feature, rendering integration mathematically impossible. For the 10X-Multiome PBMC dataset, we strictly followed the Reviewer's recommendation and downsampled from 100% to 10%, 5%, and even 1%.

The results, visualized in the newly added **S9 Fig**, demonstrate that MMIHCL avoids catastrophic collapse even under extreme sparsity. Remarkably, on the 10X-Multiome PBMC dataset with only 1% linked features, MMIHCL retained the vast majority of its full-linkage overall performance. This empirically validates that our method is robust enough to handle the "weak linkage" scenarios defined in our study.

Changes in Manuscript 1:

Firstly, we have included a new supplementary figure, **S9 Fig**, to visually demonstrate the comprehensive hyperparameter analysis. This figure follows a matrix layout where rows correspond to the four key hyperparameters (T, k, λ, and p_{link} ratio) and columns represent the three evaluation metrics (S_{bio}, S_{batch}, and S_{overall}). The bottom row specifically visualizes the robustness tests, plotting the performance trajectories of both datasets as the linkage ratio (ρ) is systematically downsampled from 100% to 10% (and further to 1% for the high-dimensional 10X-Multiome PBMC dataset).

Secondly, we have added a new **Analysis on sensitivity and robustness** subsection in the **Results** section to provide a specific quantitative interpretation of the trends observed in **S9 Fig**. The newly added text explicitly references the data shown in the bottom row of **S9 Fig** to validate the model's stability under extreme sparsity.

- **On Performance Retention:** We interpret the high S_{overall} scores plotted in the figure to prove that the model avoids catastrophic collapse (Page [22], Line [438-440]): "On the 10X-Multiome PBMC dataset with only 1% linked features, S_{overall} remained at 0.6547, retaining over 73.30% of the full-linkage performance."
- **On Alignment Stability:** We further analyze the batch correction metric from the figure to confirm robust alignment capabilities (Page [22], Line [440-442]): "Crucially, S_{batch} remained high at 0.8996 even at $\rho = 1\%$, confirming that MMIHCL can effectively leverage extremely limited shared information to guide alignment."

Comment 2:

The authors propose an adaptive kNN (akNN) strategy (Stage 1) to address the challenge of unbalanced cell numbers across different cell types. The core premise is that the number of neighbors k should be dynamically adjusted for each cell based on local density, rather than using a fixed global k. While the ablation study (Table 1) indicates that removing akNN drops performance, there is no direct analysis showing how the adaptation behaves. To validate that the akNN is functioning as intended (i.e., adapting to cell density and cluster size), I recommend the authors provide a boxplot or histogram showing the distribution of the learned neighbor counts k for each cell grouped by cell type to better demonstrate the ability of this strategy.

Response 2:

We sincerely thank the Reviewer for this insightful and constructive suggestion. We fully agree that in our original submission, merely demonstrating a performance drop in the "w/o akNN" configuration provided only indirect evidence and was insufficient to explicitly explain how the adaptation mechanism behaves in response to cell density. To rigorously validate that the akNN module functions as intended, we have implemented a comprehensive visualization analysis scheme that goes beyond the initial request:

- **Statistical Distribution (Boxplot & KDE):** We extracted the learned neighbor counts (k) for each cell and grouped them by cell type. Following the Reviewer's recommendation, we generated boxplots to display the statistical range of k values. Furthermore, while we initially attempted to use histogram as suggested, we found that the bars for different cell types were heavily overlapped in the middle range, making the distinct distributions difficult to interpret visually. Therefore, we upgraded this visualization to the Kernel Density Estimation (KDE) plot (with independent normalization). This approach offers a much clearer view of the distribution shapes and peak positions for each cell population.
- **Correlation Analysis:** We quantified the adaptive behavior by calculating the Spearman's Rank correlation (R_S) between the cluster size and the assigned k values, proving a statistically significant positive relationship.
- **Visual Verification (UMAP):** To confirm that this dynamic k assignment effectively preserves biological identity, we added a UMAP visualization annotated with cell proportions. This visually confirms that rare cell types (assigned smaller k) maintain clear boundaries without being over-smoothed by dominant populations.

This multi-faceted analysis conclusively demonstrates that our strategy successfully adapts to local density—assigning smaller neighborhoods to rare types to preserve topology and larger neighborhoods to abundant types to enhance connectivity. We have incorporated these results into the revised **Ablation study** subsection.

Changes in Manuscript 2:

To address the Reviewer's comment regarding the behavior of the akNN strategy, we have substantially expanded the **Ablation study** subsection. Following the quantitative comparison of model variants, we added a dedicated analysis focused on how the neighbor count k is dynamically adjusted. Specifically, we have introduced a new composite figure, **Fig. 8** (Page [20]), using the CITE-seq PBMC dataset as a representative example to illustrate the mechanism:

- **Fig. 8a** illustrates the severe class imbalance inherent in the data (e.g., DC vs. Mono), highlighting the necessity for adaptive neighborhood selection.
- **Fig. 8b** presents boxplots of the learned k values across cell types (derived from an initial $k = 30$). It reveals a strong positive Spearman's Rank correlation ($R_S = 0.9386$) between cluster size and k, confirming that the model automatically assigns smaller neighborhoods to rare cell types and larger neighborhoods to abundant types.
- **Fig. 8c** employs the Kernel Density Estimation (KDE) (upgraded from the standard histogram for clarity) to visualize the distribution of k values. The distinct, staggered peaks demonstrate that the model learns specific structural requirements for different populations.
- **Fig. 8d** provides a UMAP visualization annotated with cell proportions, visually verifying that this adaptive strategy successfully maintains distinct boundaries for rare clusters without over-smoothing.

To demonstrate generalizability, we extended the analysis to seven additional datasets in the newly added **S8 Fig**. By constructing adaptive graphs using the primary modality, we visualized cell type proportions alongside learned k distributions. The results consistently show a significant positive (R_S) between cluster size and neighbor counts across diverse weak and strong linkage scenarios. These findings confirm the universal effectiveness of the akNN strategy in adapting class imbalance problem.

Comment 3:

The manuscript currently relies on four metrics (ACC, FOSCTTM, GC, and ARI) to validate the performance of MMIHCL. While these provide an initial view of matching accuracy and cluster purity, they do not fully capture the nuanced trade-off between biological conservation (preserving distinct cell states) and modality alignment (mixing the datasets). To align this study with current state-of-the-art standards in single-cell computational biology, I recommend expanding the evaluation to include a broader suite of metrics established in recent benchmarking studies. Specifically, I recommend the authors refer to recent published benchmarks on diagonal and mosaic integration (e.g., `https://www.nature.com/articles/s41592-025-02856-3` and `https://www.nature.com/articles/s41592-025-02737-9`).

Response 3:

We are deeply grateful to the Reviewer for this highly insightful and constructive suggestion. We recognize the critical importance of adopting a comprehensive, multi-dimensional, and standardized evaluation framework to benchmark MMIHCL against the field's highest standards.

The seminal benchmarking studies recommended by the Reviewer (specifically the recent Nature Methods publications on diagonal and mosaic integration) provided us with invaluable guidance in refining our evaluation logic. We agree that capturing the nuanced trade-off between biological conservation and modality alignment is the key to a robust integration method. Inspired by the Reviewer's expert advice, we have significantly upgraded our evaluation system:

- **Comprehensive Expansion of the Evaluation Framework:** We have transitioned from the initial 4-metric set to a standardized 7-metric suite, strictly adhering to the protocols established in the scIB benchmark. This expanded framework now rigorously

covers three essential dimensions: **Matching Accuracy** (ACC, FOSCTTM), **Biological Conservation** (NMI, ARI, $\mathrm{ASW_{label}}$), and **Multimodal Alignment** (GC, $\mathrm{ASW_{batch}}$).
- **Holistic Performance Assessment:** To quantitatively balance biological fidelity (including matching accuracy and conservation) and batch effect removal (corresponding to multimodal alignment), we introduced a nested overall score ($\mathrm{S_{overall}}$), ensuring our method is evaluated holistically rather than by isolated metrics.
- **Enhanced Mathematical Rigor:** Recognizing the importance of reproducibility, we have added rigorous mathematical definitions and normalization details (e.g., indicator function for FOSCTTM, boundary clipping for ARI, and normalization for $\mathrm{ASW_{label}}$,) for all seven metrics in **S1 File**.

We believe that this comprehensive overhaul, prompted by the Reviewer's guidance, has significantly strengthened the validity and impact of our study. The updated and more nuanced visualized experimental results further demonstrate MMIHCL's robustness under this more stringent evaluation standard.

Changes in Manuscript 3:

To strictly adhere to the recommended benchmarking standards and provide a more rigorous assessment, we have implemented specific changes in three aspects.

Firstly, the original **Evaluation metrics** subsection, which previously comprised only brief introductory text and mathematical formulas, has been completely reconstructed in the revised **Main Text** (Page [34-35], Line [711-745]):

- **New Evaluation Framework:** The subsection has been restructured to define the seven-metric suite and the specific calculation logic for the overall score ($\mathrm{S_{overall}}$).
- **Definition of Overall Score:** We added Eq (13), which mathematically defines the weighted aggregation of biological fidelity, batch effect removal, and overall scores.
- **Explicit Categorization Logic:** The detailed categorization logic (matching vs. conservation vs. mixing) is explicitly outlined in this subsection.

Secondly, we added a new section **Details of evaluation metrics** in **S1 File** (Page [6-8], Line [202-250]). To streamline the main text, this newly added section provides rigorous mathematical definitions and explicit normalization details, such as indicator function ($\mathbb{I}_{\mathrm{paired}}$) for adaptive biology fidelity scoring, boundary conditions (e.g., clipped $\mathrm{ARI} = \max(0, \mathrm{ARI}) \in [0, 1]$), and normalization procedures (e.g., scaled $\mathrm{ASW} = (1 + \mathrm{raw\ ASW})/2 \in [0, 1]$), for all seven metrics.

Thirdly, utilizing this expanded framework, we have completely updated the experimental results presentation to provide more nuanced and comprehensive visualizations of the model's performance. Taking the results on performance analysis of weak linkage datasets (revised **Fig. 2**, Page [8]) as a representative example, we have established a new standardized visual protocol that includes three distinct layers of analysis:

- **(a) Holistic Benchmarking:** We added boxplots displaying the distribution of the new weighted overall score ($\mathrm{S_{overall}}$), providing a statistical overview of the integration quality across multiple runs.
- **(b) Definition of Overall Score:** We introduced scatter plots visualizing the balance between biological fidelity ($\mathrm{S_{bio}}$) and batch effect removal ($\mathrm{S_{batch}}$), equipped with 95%

confidence ellipses to highlight the stability and robustness of MMIHCL.

- **(c-g) Multi-dimensional Profiling:** We replaced the comprehensive comparisons with radar charts covering all seven metrics (ACC, 1-FOSCTTM, NMI, ARI, ASW_{label}, GC, and ASW_{batch}), offering a detailed "fingerprint" of method performance across different technologies.

This standardized visual logic has been consistently applied to all quantitative analyses throughout the revised manuscript, and the corresponding textual descriptions in the **Results** section have been extensively rewritten to interpret these multi-dimensional results.

Comment 4:

The manuscript currently evaluates the ability of the learned joint embeddings primarily through clustering. Since MMIHCL generates a unified, batch-corrected embedding space for inputs from different modalities, it would be compelling to test the predictive capacity of these embeddings. I recommend adding an experiment on a paired dataset (e.g., CITE-seq) to evaluate cross-modal feature prediction (such as training a regression to predict the masked omics layer using embedding).

Response 4:

We sincerely thank the Reviewer for this insightful and constructive suggestion. We fully agree with your perspective that relying solely on clustering metrics might not fully capture the fidelity of the learned joint embeddings. Validating whether the embeddings can support accurate cross-modality feature prediction is indeed a more rigorous "gold standard" for assessing whether the integration has preserved the underlying biological connections between modalities.

Following your advice, we have significantly expanded our evaluation to include quantitative feature prediction tasks. To ensure a comprehensive assessment, we extended this validation beyond the standard CITE-seq PBMC dataset (RNA $\rightarrow$ Protein) to include the more challenging 10X-Multiome PBMC dataset (ATAC $\rightarrow$ RNA), which involves predicting gene expression from sparse chromatin accessibility data. Regarding the implementation strategy, we benchmarked MMIHCL against six tools (used in the previous performance assessment) representing three distinct strategies inherent to this field:

- **Domain aggregation method** (Seurat) identify cross-modality anchors to quantify cell similarity, generating predictions via weighted averaging of features based on these anchors.
- **Imputation-based methods** (including UniPort, scConfluence, and CellLink) utilize built-in imputation capabilities driven by optimal transport plans.
- **Matching-based methods** (including MARIO, MaxFuse, and MMIHCL) share a "regression-free" paradigm, leveraging explicit cell matching to directly project features from matched counterparts.

We specifically adopted the "regression-free" explicit matching strategy (the third category) for MMIHCL. Unlike training an external regression model on top of the embeddings, which might mask alignment imperfections through the fitting of the regressor, our approach directly projects high-dimensional features from the reference to the query cells based on the learned cell-cell matching matrix. This approach relies on identifying biologically true

correspondences rather than merely aligning statistical distributions. Consequently, if the inferred cell matching is incorrect, the projected features will deviate from the ground truth. Therefore, the concordance of this matching-based prediction serves as a direct validation of whether MMIHCL has successfully established the correct biological linkages between single cells across modalities.

In the revised manuscript, we have added a new subsection titled **MMIHCL enables accurate cross-modality feature prediction via explicit cell matching** to present these findings. This section conceptually introduces the benchmarking of these three strategies and provides a detailed quantitative (PCC, CDF) and qualitative (heatmap, UMAP) analysis. The results demonstrate that MMIHCL achieves superior predictive concordance compared to aggregation-based methods and performs competitively with or surpasses other matching-based and imputation-based tools, particularly in recovering lineage-specific markers in challenging cross-regulatory tasks. Furthermore, to ensure reproducibility, we have updated **S1 File** to include the detailed implementation protocols for the cross-modality prediction tasks across all 6 benchmarked methods.

Changes in Manuscript 4:

Firstly, we have introduced a new subsection titled **MMIHCL enables accurate cross-modality feature prediction via explicit cell matching** in the **Results** section. This section presents a comprehensive evaluation of MMIHCL's predictive capacity against methods representing three distinct integration strategies.

Secondly, we included a comprehensive quantitative and qualitative evaluation on the CITE-seq PBMC dataset (as shown in **Fig. 6**, Page [14]) to demonstrate the model's cross-modality predictive capacity, with detailed panels illustrating different performance metrics.

- **Violin plots (Fig. 6a)** illustrate that MMIHCL achieves a median Pearson Correlation Coefficient (PCC) of 0.9263 in predicting protein abundance from gene expression, outperforming leading competitors such as MaxFuse (0.9213, $P < 0.001$) and CellLink (0.8840, $P < 0.001$).
- **CDF curves (Fig. 6b)** demonstrate that MMIHCL consistently encompasses the largest Area Under Curve (AUC) of 0.8775, indicating a higher proportion of cells with superior predictive correlations compared to all other baseline methods.
- **Side-by-side heatmaps (Fig. 6c)** confirm that MMIHCL faithfully recovers the z-scored expression of representative surface proteins (e.g., CD3, CD19) across annotated cell types, preserving high-frequency biological signals without the systematic underestimation of CD11c on DC as seen in MaxFuse.
- **UMAP visualizations (Fig. 6d)** further corroborate that MMIHCL accurately reconstructs the expression patterns of lineage-specific markers, maintaining the topological structure of the biological manifold between ground truth and predicted profiles.

Thirdly, we conducted a robustness evaluation on the more challenging 10X-Multiome dataset (as shown in **S7 Fig**) to test the model's generalization capabilities in a cross-regulatory context.

- **Quantitative analysis (S7 Fig a, b)** confirms that MMIHCL maintains high predictive concordance in this sparse setting. It achieved a median PCC of 0.7197 (ranking second), statistically comparable to the top-performing MARIO (0.7210, $P \geq 0.05$)

and significantly superior to scConfluence (ranking third, 0.7158, $P < 0.05$). Similarly, MMIHCL's AUC (0.7061) is comparable to the first-ranked MARIO (0.7079) and notably outperforms the third-ranked scConfluence (0.7022).

- **Qualitative inspections (S7 Fig c, d)** demonstrate that while MMIHCL performs comparably to leading methods like MARIO in general lineage marker recovery, it achieves higher fidelity in specific instances, such as preserving *SLC4A10* in MAIT cells, where MARIO exhibits distinct signal dropout. Notably, UMAP visualizations demonstrate that MMIHCL accurately reconstructs the high-expression regions of *CST3*, capturing localized high-intensity signals and preserving topological structure well.

Finally, we significantly expanded the **Methods for benchmarking** section in **S1 File** to ensure the transparency and reproducibility of our comparative analysis. We added detailed implementation protocols regarding how each of the six benchmarked methods (Seurat, UniPort, MARIO, and others) specifically performs cross-modality feature prediction.

Comment 5:

The author should check and correct the typos across the manuscript, such as in L103-104, "MMIHCL achieves the highest ACCuracy (ACC) on all datasets with an average of 85.46%, surpassing MaxFuse and MMIHCL by 1.2% and 3.72%, respectively." I think it should be "surpassing MaxFuse and" another method, not MMIHCL.

Response 5:

We sincerely apologize for this typographical issue. We agree that the original sentence was logically incorrect due to the typo. We are grateful to the Reviewer for the careful reading.

However, we would like to clarify that in the revised manuscript, the entire paragraph containing this sentence has been removed. This substantial revision was driven by the Reviewer's constructive suggestion in comment 3, which prompted us to completely reconstruct the quantitative analysis section using a more robust evaluation framework ($S_{overall}$). Consequently, the original text containing the typo has been replaced by the updated, comprehensive analysis.

Changes in Manuscript 5:

The original sentence containing the typo has been removed as a part of the rewriting of the **MMIHCL shows superiority on the weak linkage dataset integration** subsection (Page [10-13], Line [197-249]).

4 Response to Reviewer #2

Comment 1:

The fusion method of local and global information transmission adopts direct summation, but the rationality of this method is not explained. There may be weight differences between local neighbor information and global high-order information, and simple summation may dilute key information.

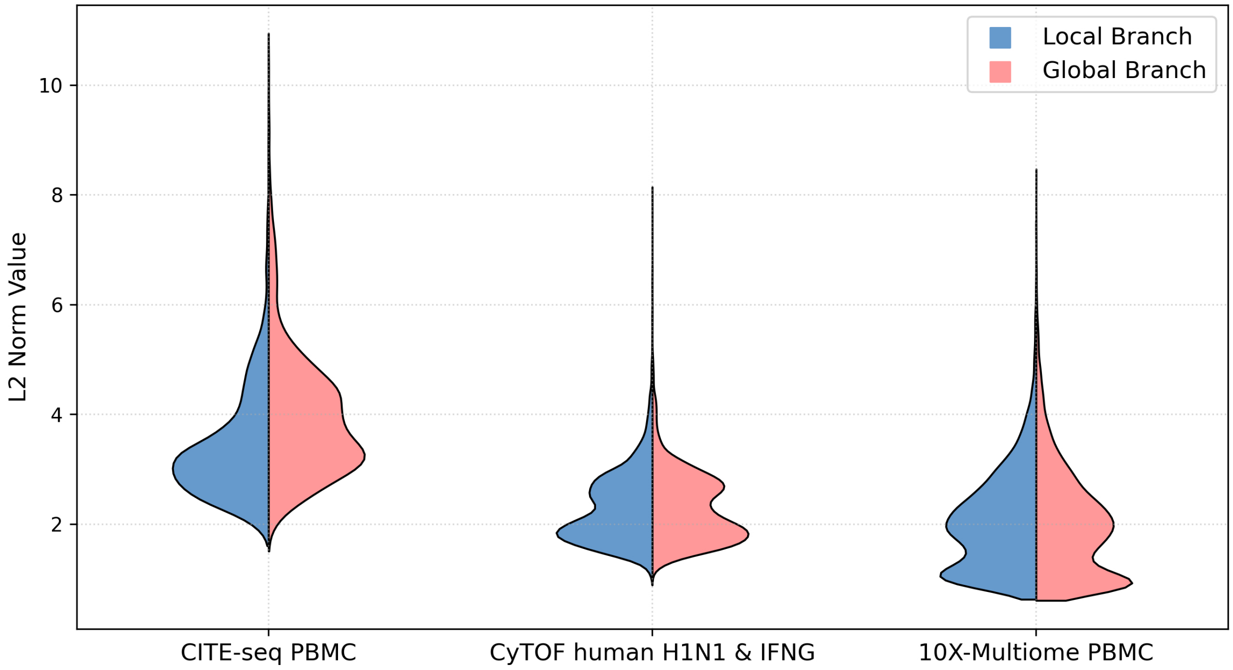

Figure R3: **Quantitative comparison of feature scales (L_2 norms) between local and global branches.** Split violin plots showing the distribution of embedding magnitudes for the local branch (Left) and global branch (Right) across three datasets.

Response 1:

We verify clearly the Reviewer's concern: if the magnitudes of the local and global representations were disproportionate, simple summation could cause the larger signal to "dilute" the smaller one. However, our extensive empirical analysis and theoretical considerations confirm that direct summation is the effective strategy for MMIHCL. We address this from two perspectives:

First, we empirically verified that the feature scales of the local and global branches are intrinsically aligned. To investigate potential "weight differences", we quantitatively analyzed the feature magnitudes of the two branches before fusion. We calculated the L_2 norm of the local neighbor and global high-order representation for every cell across three diverse datasets (CITE-seq PBMC, CyTOF human H1N1 & IFNG, and 10X-Multiome PBMC). These datasets were specifically chosen to represent three distinct modality combinations (RNA & protein, protein & protein, and RNA & ATAC), ensuring the generalizability of our findings across diverse biological contexts. As illustrated in **Fig.** R3, the feature scales of both branches are naturally aligned. In all three datasets, the distributions of L_2 norms for local and global views exhibit high visual overlap and span the same dynamic range (typically 2-8). The results confirm that the two representations operate on the same order of magnitude. Therefore, neither view numerically dominates the other, and direct summation effectively preserves the structural information from both sources without dilution.

Second, we justify our design based on the contrastive learning mechanism and the principle of parsimony. Our choice of 1:1 summation is grounded in the underlying mechanism of our model and scientific design principles:

- **Scale Alignment via Contrastive Learning:** We attribute the observed scale consistency to the contrastive learning mechanism. The core objective of MMIHCL is to maximize the agreement between the local and global views of the same cell. This optimization process enforces a strong coupling between the two branches, implicitly regularizing their feature scales to be comparable within the shared embedding space.
- **Principle of Parsimony (Occam's Razor):** Given that the feature scales are naturally balanced by the contrastive objective, introducing learnable weights (e.g., $\alpha\bar{\boldsymbol{e}}_{\boldsymbol{i}}^{(\boldsymbol{l})} + (1-\alpha)\tilde{\boldsymbol{e}}_{\boldsymbol{i}}^{(\boldsymbol{l})}, \alpha \in (0,1)$) becomes redundant. Adhering to the principle of parsimony, we avoid adding unnecessary hyperparameters, which helps reduce model complexity and mitigate the risk of overfitting on sparse single-cell data.

We have revised the **Materials and methods** section to articulate this rationale. The updated text explicitly highlights that the contrastive learning framework promotes feature magnitude alignment, justifying our use of a parameter-free summation strategy.

Changes in Manuscript 1:

We have added the corresponding theoretical rationale in the **Stage 2: hypergraph representation learning and initial matching** subsubsection following Eq (9) (Page [29-30], Line [623-631]) :

"We adopt direct summation based on two considerations. First, the local and global representations are structurally complementary. Crucially, the contrastive learning part explicitly aligns these views in a shared latent space. By minimizing the contrastive loss ($\mathcal{L}_c$), the model is driven to harmonize the feature distributions, which naturally balances the magnitudes of the local and global embeddings. Second, adhering to the principle of parsimony (Occam's razor), we employ unweighted summation as a robust, parameter-free mechanism. This avoids introducing additional hyperparameters for weight tuning, thereby reducing model complexity and mitigating overfitting risks on sparse single-cell data."

Comment 2:

During the iterative process, the subjectively defined algebraic T cannot guarantee optimal performance. Whether the desired outcome is achieved before the T-round or remains unfulfilled during the T-round.

Response 2:

We appreciate the Reviewer for raising this critical point. We agree that arbitrarily defining the iteration round T without empirical validation is insufficient to guarantee optimal performance or ensure convergence.

To rigorously determine the optimal T, we performed a sensitivity analysis by varying T from 0 to 5. We selected the CITE-seq PBMC and CyTOF human H1N1 & IFNG datasets to represent typical weak and strong linkage scenarios, respectively. This selection ensures that our chosen T is robust across varying degrees of feature overlap. The results are presented in the top row of the newly added **S9 Fig**:

- **Initial Leap ($T = 0 \to 1$):** We observed a substantial performance leap from the non-iterative baseline ($T = 0$) to the first iteration ($T = 1$), confirming that the desired outcome is indeed "fulfilled" by the iterative process.

- **Saturation Point ($T = 3$):** The performance gains saturate around $T = 3$, where the model approximately achieves peak S_{bio} scores (0.8483 for CITE-seq PBMC and 0.8589 for CyTOF human H1N1 & IFNG).
- **Overfitting Risk ($T > 3$):** Extending optimization beyond 3 rounds yields negligible improvements or even slight performance declines (potential overfitting).

Thus, $T = 3$ is not a subjective choice but an empirically determined "sweet spot" that optimally balances convergence, overall performance, and computational efficiency.

Changes in Manuscript 2:

Firstly, we have included a new supplementary figure, **S9 Fig**, where the top row specifically illustrates the performance trajectory (involving three evaluation metrics S_{bio}, S_{batch}, and $S_{overall}$) of the model as the iteration round T increases from 0 to 5. This visualizes the convergence behavior and justifies the selection of $T = 3$.

Secondly, We added a new subsection **Analysis on sensitivity and robustness** in the **Results** section to explicitly discuss the rationale behind selecting $T = 3$, citing the empirical evidence from **S9 Fig**:

- **On Baseline and Necessity of Iteration:** We incorporated a definition of the non-iterative baseline ($T = 0$) to demonstrate the immediate impact of the iterative strategy (Page [21], Line [412-416]): "Considering the iterative optimization rounds (T), it is important to note that the performance at $T = 0$ serves as a baseline... The results indicate a substantial performance leap from $T = 0$ to 1, confirming the necessity of our iterative strategy."
- **On Optimality and Convergence of Iteration:** We added an explanation regarding the saturation of performance gains to justify $T = 3$ as the empirically derived optimal stopping point (Page [21], Line [416-422]): "The gains saturate around $T = 3$... Since extending optimization beyond this point ($T > 3$) yields negligible improvements or even slight overfitting, we set $T = 3$ as the default to balance efficiency and performance."

Comment 3:

The paper's visualizations and table layouts are poorly positioned. The weak linkage datasets and strong linkage datasets should be placed in more prominent locations for better readability.

Response 3:

Regarding the layout issue, We sincerely appreciate this feedback regarding the presentation of our manuscript. Upon reviewing the original submission, we acknowledge that the previous layout had significant shortcomings that hindered the reading experience:

- **Low Information Density:** The extensive use of repetitive boxplots occupied excessive space while conveying limited insights, diluting the key takeaways.
- **Low Reading Fluency:** Figures were often clustered in the benchmarking part, forcing readers to flip back and forth between text and distant figures.

In the revised manuscript, we have completely overhauled the visualization strategy to achieve a "comprehensive layout upgrade":

- **Elevate Information Density:** We replaced the scattered single-metric plots with high-density composite figures. For example, in **Fig. 2**, we integrated multiple statistical visualization types (including multi-dimensional metric summaries and trade-off analyses) into a unified panel. This allows readers to grasp the comprehensive performance profile within a single visual unit, significantly reducing cognitive load.
- **Promote Logical Fluency:** We rigorously aligned the placement of figures with the narrative flow. Visualizations now appear immediately adjacent to the corresponding analysis. For instance, the cross-modality prediction performance is visually anchored by **Fig. 6**, and the disease stratification findings are instantly supported by **Fig. 7**. This ensures that visual evidence is always available exactly when the reader needs it to verify the textual claims.

Furthermore, regarding the Reviewer's suggestion to emphasize the distinction between weak and strong linkage datasets, we completely agree with the Reviewer that the distinction between weak and strong linkage datasets is fundamental to the readability and impact of this study. This point aligns with the Editor's suggestion regarding the need for a rigorous definition of linkage intensity. To address this, we have enhanced the prominence and clarity of these categories through a more systematic approach:

- **Quantitative Framework:** We have introduced a formal quantitative metric to replace qualitative descriptions.
- **Explicit Labeling:** All datasets and experimental scenarios have been explicitly labeled as "weak linkage" or "strong linkage" throughout the **Results** and **Materials and methods** sections.
- **Structural Integration:** We have restructured the logical flow to ensure that these quantitative definitions are prominently positioned and consistently cross-referenced.

We believe these modifications effectively highlight the different linkage scenarios as requested, significantly improving the manuscript's overall readability.

Changes in Manuscript 3:

Regarding the layout issue, we present some representative examples of figures and tables to illustrate how the revised layout logic has been concretely implemented:

- **High-Density Visualization (Fig. 2, Page [8]):** We consolidated scattered plots into unified, high-density panels. **Fig. 2** now integrates radar charts (displaying the balance of six metrics), bio-vs-batch scatter plots, and aggregated boxplots into a single view. This layout allows readers to efficiently assess MMIHCL's comprehensive performance profile—covering biological fidelity and multimodal alignment—without flipping through multiple pages of redundant charts.
- **Synchronized Text-Figure Flow (Fig. 7, Page [16]):** We repositioned all major figures to tightly synchronize with the narrative, eliminating the need for frequent page-turning. In the new subsection **MMIHCL empowers robust disease classification and drug target discovery**, **Fig. 7** is positioned close to the relevant text. As the reader processes the quantitative claims about T1D separation, they can instantly glance at the adjacent **Fig. 7a** (UMAP) for confirmation; similarly, discussion of gene markers like CXCL10 is immediately supported by the **Fig. 6c** (violin plot) and **Fig. 7c** (volcano plot).

- **Hierarchical Table Organization (Table 1, Page [19] & S1 Table):** To reduce visual clutter caused by stacking homogenous tables, we implemented a hierarchical structure. We condensed the most critical quantitative indicators from the ablation experiments into a concise **Table 1** in the main text for high-level summary, while moving the voluminous, detailed metric breakdowns to **S1 Table**. This ensures the main text remains streamlined while maintaining rigor for interested readers.

Furthermore, we have formalized the quantitative criteria for distinguishing between weak and strong linkage scenarios within the methodological framework. This update ensures that the datasets used in our study are categorized based on a rigorous, objective standard rather than qualitative descriptions.

- **Quantitative Framework:** We have introduced the mathematical definition of the linkage ratio (ρ) to provide a consistent baseline for linkage intensity across different omics. As stated in the revised **Materials and methods** section (Page [26], Line [539-540]): "To quantitatively measure the linkage intensity between modalities, we define the linkage ratio ρ for each dataset as: $\rho = \min(p_{link}/p_x, p_{link}/p_y)$. (1)"
- **Explicit Labeling:** We have established clear numerical thresholds to categorize datasets, moving away from subjective terminology. The revised text in **Materials and methods** section (Page [26], Line [542-545]) now specifies: "A scenario is defined as weak linkage if the linked features represent a small fraction of at least one modality, i.e., $\rho < 10\%$. Conversely, a scenario is defined as strong linkage if both modalities share a substantial portion of linked features, i.e., $\rho \geq 10\%$."
- **Structural Integration:** These quantitative criteria have been systematically integrated throughout the manuscript to guide the presentation of results. For instance, in the **Results** section, we have updated the descriptions to reflect this new standard: "We categorize the linkage intensity between two modalities based on the linkage ratio ρ defined in Eq (1). Specifically, two datasets are defined as weakly linked if $\rho < 10\%$." (Page [5], Line [136-137]) Similarly for strong linkage, we state "We also tested whether MMIHCL shows competitive performance on strongly linked modalities, which, according to the criteria in Eq (1), are defined as scenarios where $\rho \geq 10\%$." (Page [10], Line [202-204])

By implementing these changes, we have ensured that the linkage intensity of each dataset is prominently displayed and mathematically grounded. This not only addresses the Reviewer's concern regarding readability, but also reinforces the theoretical robustness of our comparative analysis across diverse single-cell sequencing technologies.

Comment 4:

In the Ablation studies section, the impact of individual modules was examined by modifying a single module to observe its effect. However, the combined effects of multiple modules might be difficult to determine. This challenge can be addressed by introducing multi-module modifications to assess their synergistic influence.

Response 4:

We sincerely thank the Reviewer for this valuable suggestion. We acknowledge that our original ablation study, which focused primarily on removing individual components one at

a time, was insufficient to fully demonstrate the systematic advantages of the MMIHCL architecture.

Accepting your advice, we have introduced a "Baseline GCN" configuration in the revised manuscript. This setup serves as a foundational reference point by simultaneously removing all the key proposed modules (Reliability Filter, akNN mechanism, and Hypergraph Contrastive Learning). By establishing this lower-bound baseline, we can now more effectively highlight the substantial combined contribution and synergistic effects of our proposed components against a standard graph learning backbone. We have updated the **Ablation study** subsection to include this comprehensive comparison.

Changes in Manuscript 4:

To comprehensively validate the combined contribution of MMIHCL's components, we have expanded the **Ablation study** subsection by adding a new configuration labeled "Baseline GCN" (Page [18], Line [362-366]). This baseline represents a conventional graph learning approach where all three core modules are removed:

- The **Reliability Filter** is completely disabled (retaining all matching pairs).
- The **akNN mechanism** is replaced by a standard kNN with a constant k.
- The **Hypergraph Contrastive Learning** is replaced by a standard GCN without dual-view contrastive alignment.

As shown in the revised **Table 1** (Page [19]), the "Baseline GCN" consistently exhibits the lowest performance across all metrics. For instance, in the weak linkage scenario, it shows a sharp decline in ACC (79.53% vs. 86.47% for Full MMIHCL) and overall score ($\mathbf{S}_{\mathbf{overall}}$ 0.7912 vs. 0.8398), with statistically significant differences ($P < 0.001$). This significant performance gap confirms that the superior performance of MMIHCL stems from the synergistic integration of its modules rather than any single component alone.

For a granular breakdown, **S1 Table** details the mean values, standard deviations, and performance differences for all 8 datasets across all 10 metrics (including $\mathrm{S}_{\mathrm{bio}}$, $\mathrm{S}_{\mathrm{batch}}$, and $\mathrm{S}_{\mathrm{overall}}$), explicitly corroborating the consistent poor performance of the "Baseline GCN" configuration.

Comment 5:

The study selected only two weak association integration methods—MaxFuse and sc-Confluence—as baselines, excluding other mainstream approaches published during the same period (including weak association integration tools introduced after 2023). This limitation may undermine the conclusion that MMIHCL achieves optimal performance' due to insufficient comparative evidence.

Response 5:

We sincerely appreciate the Reviewer for this highly constructive observation. We fully recognize that the field of single-cell multimodal integration is advancing rapidly, and incorporating the recent SOTA baselines is essential to establish the robustness and timeliness of our study. Your suggestion to broaden the comparative landscape has significantly strengthened the evaluation framework of our work.

To comprehensively address this concern and ensure our benchmarking represents the current cutting-edge, we have expanded our experimental scope to include **CelLink** (bioRxiv,

Nov 2024), a newly introduced method specifically designed for weak-linkage integration. Our selection of CelLink was driven by a consensus of expert opinions. Firstly, it directly fulfills your requirement for a mainstream tool published after 2023. Secondly, it aligns with the specific and explicit recommendation provided by the Reviewer #3 (comment 1). By integrating this latest method, we aim to provide a more rigorous and convincing assessment that satisfies the high standards set by the Reviewers.

Changes in Manuscript 5:

In direct response to the Reviewer's feedback regarding the need for SOTA baselines, we have extensively revised the manuscript to incorporate **CelLink**. This integration is not merely an added experiment but a comprehensive update spanning the theoretical contextualization in the **Introduction** section, to the rigorous benchmarking in the **Results** section, ensuring that MMIHCL is evaluated against the recent advancements in the field.

Firstly, we have revised the **Introduction** section to explicitly acknowledge CelLink's contribution to the field. As shown on Page [3-4], Line [77-82], we added the description: "...several methods such as scConfluence, MaxFuse, and the recently developed CelLink, have recently been developed... Specifically, CelLink addresses the issues of weak feature correlation and imbalanced cell populations through an iterative unbalanced optimal transport framework." This ensures that our study is positioned relative to the latest "after 2023" methodologies. We also have updated the baselines information in the **Results** section to include CelLink as a standard comparator. As stated on Page [5-6], Line [138-141]: 'We used six methods published for multimodal integration as baselines, including... and CelLink." Furthermore, the detailed hyperparameter settings and implementation protocols for CelLink have been added to the **Methods for benchmarking** section in **S1 File** (Page [2-3], Line [62-83]) to ensure reproducibility.

Secondly, in terms of quantitative assessment, we have rigorously updated the benchmarking analysis on weak linkage datasets to explicitly compare MMIHCL against CelLink, providing concrete statistical evidence of MMIHCL's sustained superiority.

- **Holistic Evaluation Update:** We have reconstructed the quantitative analysis in the **Results** section to benchmark MMIHCL against CelLink using our upgraded overall evaluation framework.
- **Specific Performance Evidence:** The updated results confirm that MMIHCL retains its optimal position. As highlighted in the revised text (Page [7], Line [162-164]): "Even compared to SOTA weak linkage integrators, such as MaxFuse ($\Delta = -0.0226$), scConfluence ($\Delta = -0.0175$), and CelLink ($\Delta = -0.0420$), MMIHCL maintains a significant lead." This data quantitatively demonstrates MMIHCL's superiority of weak association integration even against the recent 2024 baseline.
- **Expanded Figures:** We have updated **Fig. 2** (Page [8]) and **Fig. 3** (Page [9]) to benchmark CelLink in typical weak linkage scenarios, explicitly including it in the statistical boxplots, performance trade-off scatter plots, multi-dimensional radar charts, and UMAP visualization, to intuitively and vividly demonstrate the robust superiority of MMIHCL. Therefore, the robust superiority of MMIHCL can be demonstrated in an intuitive and vivid manner.

Comment 6:

This study only evaluated computational costs under a single-server setup without comparing runtime and memory consumption across varying cell populations (e.g., 1k, 10k, 100k cells), thus failing to demonstrate the efficiency advantage of MMIHCL on large-scale datasets.

Response 6:

We sincerely thank the Reviewer for this constructive suggestion. We apologize for the lack of scalability analysis in the previous version and fully agree that evaluating computational costs across varying cell population sizes is crucial for demonstrating the method's efficiency on large-scale datasets.

In response to this comment, we have expanded our computational cost assessment by conducting a scalability analysis on datasets ranging from 10k (the original size of the CITE-seq PBMC dataset) up to 100k cells (generated via bootstrap sampling). We recorded both Running Time (RT) and Memory Consumption (MC) for MMIHCL and baseline methods at each scale.

Changes in Manuscript 6:

We have extensively revised the manuscript to incorporate these scalability assessments:

- **Specification of Experimental Design:** We first clarified the experimental protocols in the **Analysis on computational cost** subsection (Page [22-24], Line [445-472]), specifying that the large-scale datasets (ranging from 10k to 100k cells) were generated via bootstrap sampling from the CITE-seq PBMC dataset (used in the previous performance assessment). We also defined the measurement standards, which involved averaging five independent runs for datasets $\leq$ 30k cells and imposing strict cutoffs (4 hours for runtime, and 64 GB for memory) to ensure a rigorous evaluation.
- **Visualization of Scalability Trends:** Based on this design, **Fig. 9c** and **Fig. 9d** (Page [23]) within the **Analysis on computational cost** subsection illustrate the resulting efficiency profiles. These figures visually plot the RT and MC curves across the expanded dataset sizes, explicitly marking threshold violations ("Timeout" and "out of Memory") for baseline methods where applicable.
- **Detailed Textual Analysis:** We updated the accompanying text in the **Analysis on computational cost** subsection to interpret these findings. The revised analysis highlights that MMIHCL maintains stable scalability and exceptional memory efficiency (consuming only 2.16 GB at 100k cells), contrasting significantly with baselines that failed due to computation time (e.g., MARIO) or excessive resource demands (e.g., CelLink).

5 Response to Reviewer #3

Comment 1:

Can the authors perform statistical tests of the calculated evaluation metrics to evaluate the significance of the performance difference across different methods? It is also better to incorporate another specialized method CelLink for comparative analysis (bioRxiv preprint

doi: https://doi.org/10.1101/2024.11.08.622745).

Response 1:

We sincerely appreciate the Reviewer for these professional and specific suggestions. We fully agree that providing statistical evidence and benchmarking against the latest specialized methods are crucial for establishing the rigor and validity of our conclusions. To address your concerns, we have implemented a two-fold major revision:

- **Addition of Statistical Tests:** We have performed rigorous statistical analyses to quantify the significance of performance differences. Specifically, we applied the two-sided Wilcoxon signed-rank tests for global performance metrics (S_{bio}, S_{batch}, and $S_{overall}$) and individual metrics (ACC, 1-FOSCTTM, NMI, and others). The results confirm that the improvements achieved by MMIHCL are statistically significant.
- **Incorporation of CelLink:** We have strictly followed your recommendation to incorporate CelLink as a core baseline in our evaluation framework. Our updated experiments demonstrate that while CelLink is a competitive method, MMIHCL consistently maintains a lead in performance across diverse datasets.

Changes in Manuscript 1:

To strictly comply with the Reviewer's constructive guidance and firmly establish the validity of our conclusions, we have executed a comprehensive revision of the manuscript.

Firstly, we have integrated a complete statistical evaluation framework into the **Results** section, specifically focusing on the weak linkage integration scenarios where performance differentiation is most critical.

- **Methodology and Visual Updates:** We have updated **Fig. 2** (Page [8]) to include rigorous significance testing. As detailed in the revised figure caption: "Statistical significance for (a) and (b) was determined by two-sided Wilcoxon signed-rank tests...", and "Significance markers for metrics indicate statistical differences between the two top-ranked methods (determined by two-sided Wilcoxon signed-rank tests).", these markers visually confirm MMIHCL's dominance across key metrics.
- **Quantitative Interpretation:** We have rewritten the quantitative analysis subsection to explicitly report these statistical outcomes. For instance, we added the statement (Page [7], Line [158-160]): "The mean $S_{overall}$ of MMIHCL consistently surpasses the distributions of all baseline methods, demonstrating a statistically significant advantage (Wilcoxon signed-rank tests, $P < 0.001$).".
- **Significance against SOTA:** We further clarified that this significance holds true even against the latest methods. As noted in the revised text (Page [7], Line [162-164]): "Even compared to SOTA weak linkage integrators, such as... CelLink... MMIHCL maintains a significant lead.".

Secondly, we have strictly followed your recommendation to incorporate **CelLink** as a core baseline. We have embedded this new method into every stage of our evaluation framework, from theoretical positioning to experimental benchmarking:

- **Methodological Updates:** We have revised the **Introduction** section to formally recognize CelLink as a SOTA weak linkage integrator in "...several methods such as scConfluence, MaxFuse, and the recently developed CelLink..."(Page [3], Line [77-80]).

Furthermore, to ensure rigorous reproducibility, we have detailed its implementation in the **Methods for benchmarking** section in **S1 File**. Notably, as described in the revised supplementary text (Page [2-3], Line [74-79]), "For the scATAC-seq data, we converted the chromatin accessibility peaks into a gene activity matrix... ensuring compatibility with CelLink's input requirements.", which demonstrates our careful handling of multimodal data structures.

- **Quantitative Benchmarking:** We have reconstructed the comparative analysis in the **Results** section to explicitly benchmark MMIHCL against CelLink. The updated results provide concrete evidence of our method's superiority. As stated in the revised **MMIHCL shows superiority on the weak linkage dataset integration** subsection (Page [7], Line [162-164]): "Even compared to SOTA weak linkage integrators, such as... and CelLink ($\Delta = -0.0420$), MMIHCL maintains a significant lead." This metric confirms that MMIHCL remains the optimal choice for weak linkage scenarios.
- **Visual Comparison:** We have comprehensively updated the visual evidence. **Fig. 2** (Page [8]) now includes CelLink in the statistical boxplots, scatter plots, and radar charts to show the performance gap. Additionally, we updated the UMAP visualizations in **Fig. 3** (Page [9]) to provide a qualitative comparison, where we analyze that "Furthermore, while advanced baselines such as... CelLink show improved integration performance, MMIHCL consistently exhibits the most distinct cell-type boundaries..." (Page [9-10], Line [191-193]).

Comment 2:

For the ablation study, the performance improvements from individual components appear minor. Could the authors add statistical tests to substantiate these findings? Furthermore, to isolate the contribution of the hypergraph architecture more precisely, it would be valuable to include a comparison where only the hypergraph learning module is replaced with a general graph learning module, while the contrastive learning component is retained.

Response 2:

We sincerely thank the Reviewer for this critical and insightful suggestion. We agree that our original ablation design (which only included the simultaneous removal of both the Hypergraph and Contrastive Learning) did not fully isolate the specific contribution of the hypergraph structure itself.

To address this and rigorously verify whether the hypergraph architecture prevents oversmoothing compared to a general graph backbone, we have implemented the suggested "w/o Hypergraph with CL" configuration. This new setup retains the contrastive learning framework but replaces the hypergraph branch with a standard graph convolution module. Furthermore, as requested, we have applied two-sided Wilcoxon signed-rank tests to all ablation comparisons in the revised **Table 1** to substantiate the statistical significance of our findings.

Changes in Manuscript 2:

To isolate the structural contribution of the hypergraph, we added a specific configuration named "w/o Hypergraph with CL" to the **Ablation studies** subsection (Page [18], Line [354-357]). In this setup, the hypergraph branch is removed, and the Contrastive Learning

(CL) scheme is modified to align the raw embeddings with standard GCN-aggregated embeddings (normal graph view) rather than aligning the standard GCN-aggregated embeddings with the hypergraph view (as in the full model).

The results reveal a critical trade-off. As discussed in the revised manuscript (Page [19], Line [372-373]): "Notably, the 'w/o Hypergraph with CL' setup exhibits a significant decline in S_{bio} ($P < 0.001$) despite a marginal increase in S_{batch}." This finding suggests that relying solely on the simple graph view "leads to over-smoothing, where distinct cell populations are merged to achieve artificial mixing at the expense of biological distinctness" (Page [19], Line [373-375]). This confirms that the dual-view architecture is essential for balancing batch correction with the preservation of biological heterogeneity.

We have added statistical significance annotations (***: $P < 0.001$, **: $P < 0.01$, *: $P < 0.05$, ns: not significant) to **Table 1** based on the Wilcoxon signed-rank tests. For a granular breakdown, **S1 Table** details the mean values, standard deviations, and performance differences for all 8 datasets across all 10 metrics (including S_{bio}, S_{batch}, $S_{overall}$, and others). These detailed results explicitly corroborate the consistent trend where the "w/o Hypergraph with CL" setup exhibits a significant decline in S_{bio} despite marginal increases in S_{batch}.

Comment 3:

The paper highlights the significance of the hypergraph learning module within the MMIHCL framework; however, the corresponding description in the methodology section lacks sufficient detail. Specifically, the methodology section notes that $\mathbf{W}$ in Equation 6 is a dynamically learnable parameter matrix encoding high-order intrinsic relationships among the n embeddings of $\mathbf{E}$. In contrast, conventional hypergraph learning typically entails the explicit construction of a hypergraph structure (e.g., each node and its K-nearest neighbors form a hyperedge), followed by the derivation of each node's embedding via an information propagation mechanism that transfers information from nodes to hyperedges and subsequently back to nodes. Could the authors provide a rationale of such a design?

Response 3:

We sincerely appreciate the Reviewer's profound insights and professional comments regarding the design of our hypergraph learning module. This constructive suggestion has been immensely helpful in allowing us to refine and clarify the scientific mechanism underlying the hypergraph-style information propagation in MMIHCL.

The rationale for employing a dynamically learnable parameter matrix $\mathbf{W}$ for implicit hypergraph construction, rather than an explicit (e.g., kNN-based) structure, is based on the following scientific considerations:

- **Adherence to the Hypergraph Propagation Paradigm:** As correctly pointed out by the Reviewer, standard hypergraph learning follows a "node-hyperedge-node" paradigm. In our framework, the term $\mathbf{H} = \mathbf{E^{(0)}W}$ is designed as a parameterized "soft" incidence matrix that maps n cells to K virtual hyperedges. The operation $\mathbf{H}(\mathbf{H}^T\mathbf{E^{(l-1)}})$ in Eq (7) precisely executes this paradigm: $\mathbf{H}^T$ aggregates cell features into the latent hyperedge space (node to hyperedge), and $\mathbf{H}$ subsequently redistributes this high-order global context back to individual cells (hyperedge to node).
- **Enhanced Flexibility for Noisy Distributions:** In scenarios involving weakly linked

modalities, initial feature correlations can be sparse or contain noise. While explicit construction typically fixes the hyperedge topology based on initial features, our learnable $\mathbf{W}$ allows the high-order relations to be refined in a data-driven manner throughout the training process. This flexibility enables the model to capture robust global dependencies that may transcend the local constraints of the initial graph.
- **Synergy between Architecture and Supervision:** This implicit design allows the formation of high-order structures to be directly driven by the contrastive loss $\mathcal{L}_c$. This ensures that while the global branch explores complex manifolds, it remains grounded in the biological identities preserved by the local branch.

We are grateful to the Reviewer for pointing out this lack of detail, which has allowed us to significantly strengthen the theoretical rigor of our methodology. We have added the corresponding rationale in the revised **Materials and methods** section.

Changes in Manuscript 3:

To provide a comprehensive rationale for the hypergraph learning module and its integration with contrastive learning, we have implemented the following textual modifications in the **Materials and methods**.

Firstly, we present the complete new paragraph regarding the rationale behind the hypergraph design following Eq (7) (Page [29], Line [605-610]):

"This design utilizes $\mathbf{H} = \mathbf{E^{(0)}}\mathbf{W}$ as a 'soft' incidence matrix mapping n cells to K virtual hyperedges. The operation$\mathbf{H}(\mathbf{H}^T\mathbf{E^{(l-1)}})$ executes the 'node-hyperedge-node' paradigm: $\mathbf{H}^T$ aggregates features into latent hyperedge space, and $\mathbf{H}$ redistributes high-order context back to cells. Dynamically learning $\mathbf{W}$ captures global dependencies transcending initial local constraints, ensuring robustness for complex, weakly linked multimodal distributions."

Secondly, we present the complete revised transition for the contrastive learning objective function (Page [29], Line [614-618]):

- **Original:** To guide the cell's local messages to supervise the learning of global messages, we next perform contrastive learning at each layer on embeddings from two branches $\bar{\boldsymbol{e}}_{\boldsymbol{i}}^{(\boldsymbol{l})}$, $\tilde{\boldsymbol{e}}_{\boldsymbol{j}}^{(\boldsymbol{l})}$ for $i, j = 1, 2, \cdots, n$ and $l = 1, 2, \cdots .L$. The contrastive learning objective loss function is as follows:
- **Revised:** Contrastive learning aligns both branches at each layer to ensure $\mathbf{H}$ captures biologically relevant dependencies. By maximizing agreement between the local (neighborhood-preserving) and global (high-order manifold) branches ($\bar{\boldsymbol{e}}_{\boldsymbol{i}}^{(\boldsymbol{l})}$, $\tilde{\boldsymbol{e}}_{\boldsymbol{j}}^{(\boldsymbol{l})}$ for $i, j = 1, 2, \cdots, n$ and $l = 1, 2, \cdots .L$), $\mathcal{L}_c$ grounds the implicit hypergraph in cell identities while exploring global structures. The objective function is defined as:

Comment 4:

The paper briefly outlines the selection criteria for hyperparameters in the experimental setup. Could the authors perform a sensitivity analysis on key hyperparameters, such as the initial k-value employed to generate the adjacent cell graph and the regularization term coefficient λ in Equation 9? Furthermore, the number of iterative optimization rounds T in Stage 3 is set to a maximum of 3—can this be attributed to the sufficiently robust initialization achieved in Stage 2?

Response 4:

We sincerely thank the Reviewer for these valuable suggestions regarding hyperparameter sensitivity. We are particularly grateful for the Reviewer's insightful hypothesis regarding the connection between the iteration rounds (T) and the initialization in Stage 2. This hypothesis coincides perfectly with our original design philosophy—that Stage 2 should provide high-quality initial matching and CCA-based embeddings to facilitate rapid convergence in Stage 3. We acknowledge that, prior to your comment, this crucial advantage was implicitly embedded in our experiments but not explicitly emphasized in the text.

To thoroughly address these concerns, we have added a new subsection titled **Analysis on sensitivity and robustness** within the **Results** section. Additionally, we have introduced a comprehensive supplementary figure, **S9 Fig**, to visually support these findings.

Following the Reviewer's recommendation, we firstly conducted a systematic grid search on the neighbor count ($k \in [5, 30]$) and the regularization coefficient ($\lambda \in [0.0001, 1]$) using both weak linkage (CITE-seq PBMC) and strong linkage (CyTOF human H1N1 & IFNG) datasets. As shown in the second and third rows of the new **S9 Fig**, MMIHCL exhibits broad stability. The integration metrics remain robust within $k \in [15, 25]$ and $\lambda \in [0.001, 0.1]$, indicating that the model effectively captures local topology and prevents overfitting without requiring exhaustive fine-tuning.

Furthermore, to address the Reviewer's specific question about the role of Stage 2 in determining $T = 3$, we followed a logical verification process:

- **Hypothesis & Verification:** Motivated by the Reviewer's hypothesis, we analyzed the performance trajectory from the non-iterative baseline ($T = 0$) to over-iterated states ($T = 5$), as visualized in the top row of **S9 Fig**. We specifically examined the performance at $T = 0$ (representing the output of Stage 2) and the subsequent gains.
- **Observation & Confirmation:** The results show that the baseline performance at $T = 0$ is already substantial, providing a solid foundation. This is followed by a significant performance leap at $T = 1$ and rapid saturation around $T = 3$. These empirical results allow us to affirmatively confirm the reviewer's hypothesis. The reason MMIHCL achieves optimal performance in just 3 rounds is indeed attributable to the robust initialization strategy (detailed in Stage 2). This "warm start" ensures that the model is already close to the optimal manifold before the iterative refinement begins, thereby significantly reducing the computational cost required for convergence. We have explicitly added this discussion to the revised manuscript.

Changes in Manuscript 4:

Firstly, we added **S8 Fig**, a comprehensive hyperparameter analysis matrix:

- The top row illustrates the convergence trajectory of iteration rounds (T) from 0 to 5, explicitly showing the significant performance leap from the baseline ($T = 0$) to the saturation point ($T = 3$), which visually justifies the stopping criterion.
- The second and third rows visualize the sensitivity analysis for the neighbor count (k) and regularization coefficient (λ), demonstrating the model's stability across a wide parameter space.

Secondly, we We added a new subsection **Analysis on sensitivity and robustness** to discuss these results.

- **On k and λ:** We added text describing their stable ranges (Page [21-22], Line [425-430]):

"...our results show that integration performance remains robust within $k \in [15, 25]$... MMIHCL maintains consistent S_{overall} scores within $\lambda \in [0.001, 0.1]$, indicating that the method effectively prevents overfitting without requiring exhaustive fine-tuning."

- **On T and Stage 2 Initialization:** We explicitly linked the fast convergence to the Stage 2 initialization, as suggested (Page [21], Line [416-420]): "The gains saturate around $T = 3$... This rapid convergence can be largely attributed to the robust initialization strategy (detailed in Materials and methods, MMIHCL pipeline, Stage 2), which provides a high-quality starting point for the subsequent optimization."

Comment 5:

Are there new biologically findings compared to other methods?

Response 5:

We appreciate this insightful question. We acknowledge that the original manuscript focused primarily on technical integration metrics and lacked a demonstration of downstream biological discovery.

To address this, we have introduced a completely new subsection, **MMIHCL empowers robust disease classification and drug target discovery**, in the revised manuscript. By conducting comparative analyses on application-specific datasets (HPAP and Kang18), we demonstrate that MMIHCL provides superior biological resolution compared to SOTA methods:

- **Sharper Disease Classification (Specificity Comparison):** In the HPAP dataset (Type 1 Diabetes vs. Control), a major challenge is distinguishing true pathological states from technical batch effects without confounding cell type identity. Compared to other methods, MMIHCL achieved the clearest separation between disease and healthy states while preserving cell type coherence. Quantitative benchmarking confirmed this new finding: MMIHCL yielded the highest Status NMI and ARI scores, indicating that it captured disease-specific latent structures that were less distinct or conflated in the embeddings generated by other tools.
- **Recovering Lost Drug Targets (Sensitivity Comparison):** In the Kang18 PBMC dataset (IFN-β stimulation), the biological signal of drug response is often subtle and masked by technical noise. Our analysis reveals that while baseline methods often over-smooth these signals or difficult to distinguish them from background noise, MMIHCL acts as a robust signal filter. Specifically, compared to other methods, MMIHCL accurately recovered the expression patterns of canonical Interferon-Stimulated Genes (ISGs) such as *CXCL10*. This allowed for the identification of potential therapeutic targets that other methods missed.

In summary, the new biological findings enabled by MMIHCL is the ability to unveil meaningful biological heterogeneity and therapeutic targets that remain hidden when using standard integration techniques.

Changes in Manuscript 5:

To highlight the new biological findings enabled by MMIHCL compared to other methods, we have made the following specific revisions.

Firstly, we added a new subsection **MMIHCL empowers robust disease classification and drug target discovery** in the **Results** section. In this subsection, we provided rigorous quantitative evidence demonstrating MMIHCL's superior biological resolution over SOTA baselines:

- **For Disease Classification (HPAP dataset):** We reported that MMIHCL achieved the most distinct separation between Type 1 Diabetes (T1D) and Control samples. Quantitatively, MMIHCL yielded the highest Status NMI (0.4618) and ARI (0.5439) compared to baselines, proving its superior ability to capture disease-specific latent structures (Page [17], Line [311-315]). Furthermore, within the T1D subpopulation, MMIHCL maintained the highest cell-type ARI (0.4632), indicating it effectively distinguishes pathological states without sacrificing the resolution of specific islet cell subtypes (Page [17], Line [315-320]).
- **For drug target discovery (Kang18 PBMC dataset):** We presented a comparative statistical analysis showing that MMIHCL recovers drug-response signals more accurately than competing methods. Specifically, for the key marker gene *CXCL10*, MMIHCL achieved the most significant statistical difference between control and stimulated groups with a P-value of 1.10×10^{-120}, significantly outperforming Seurat ($p = 6.82 \times 10^{-119}$) and MARIO ($p = 2.60 \times 10^{-112}$) (Page [17], Line [325-331]). This superior signal-to-noise ratio enabled the identification of both drastic effectors (e.g., *LIMK2* and *CEBPD*) and subtle regulators (e.g., *ANO9* and *CXCL10*) that might be missed by other methods (Page [17-18], Line [332-338]).

Secondly, we introduced a new multi-panel **Fig. 7** (Page [16]) to visually present these comparative findings:

- **Fig. 7a** visualizes the joint embeddings of the HPAP dataset. The top row (colored by disease status) clearly shows that MMIHCL achieves a cleaner separation between disease and control states compared to the mixed or less distinct clusters observed in Seurat and MaxFuse, visually confirming the quantitative metrics. Crucially, the bottom row (colored by cell type) confirms that this disease separation does not come at the cost of biological identity. MMIHCL preserves distinct clusters for major cell types just as well as, or better than, the baselines.
- **Fig. 7b** is particularly relevant to the comparison of biological findings. It displays split violin plots for representative ISGs like *CXCL10*, *CXCL11*, and *CCL2*. These plots directly juxtapose MMIHCL against Seurat and MARIO, visually demonstrating that MMIHCL-aggregated profiles exhibit the sharpest peaks and clearest separation between conditions, effectively denoising the biological signal where other methods show more similar distributions.
- **Fig. 7c** illustrates the broad dynamic range of therapeutic targets identified by MMIHCL from the smoothed expression profile. The plot reveals a wide spectrum of significant gene expression changes, with Log2 Fold Changes ranging from -6.28 to +5.14 and statistical significance (-Log10 P-value) reaching exceeding 120. It highlights MMIHCL's sensitivity in capturing a broad spectrum of targets, distinguishing drastically altered effectors from subtly regulated but highly significant markers.

Comment 6:

This part including 3 minor comments:

1. Line 104: surpassing MaxFuse and MMIHCL → surpassing MaxFuse and scConfluence.

2. S6 Fig: The UMAP subgraphs for scConfluence—colored by data modality and cell type—should be adjusted to ensure consistency.

3. The method names on Fig. 3 and Fig. 4 are two big and please adjust it to match the font size of other texts.

Response 6:

We sincerely apologize for these issues including incorrect spellings, inconsistent graphics, and oversized fonts. We sincerely thank the Reviewer for these constructive minor comments, which have helped improve the text accuracy, visual clarity, and consistency. We are grateful to the Reviewer for the careful reading of the main text and supplementary materials.

Regarding minor comment 1, we appreciate the Reviewer pointing out this error We note that this issue was also highlighted by Reviewer #1 in his comment 5. We agree that the original sentence was logically incorrect. However, as detailed in our response to the Reviewer #1, the entire paragraph containing this sentence has been removed in the revised manuscript to accommodate the comprehensive reconstruction of the quantitative analysis section. Consequently, this typo is no longer present.

We have also carefully addressed minor comments 2 and 3 to ensure visual clarity and consistency.

Changes in Manuscript 6:

Firstly, the original paragraph containing this typo has been removed from the **MMIHCL shows superiority on the weak linkage dataset integration** subsection (Page [10-13], Line [197-249]) due to the reconstruction of the quantitative analysis framework.

Secondly, we have adjusted the UMAP subgraphs for scConfluence in revised **S4 Fig** (original **S6 Fig**) to ensure that the upper part is colored by data modality, and the lower part is colored by cell type (as shown in Fig. R4 for quick comparison). This ensures the consistency of the layout logic for all UMAP subgraphs.

Thirdly, we have reduced the font size of the method names in revised **Fig. 3** (Page [9], original **Fig. 3**) and **5** (Page [12], original **Fig. 4**) to match the font size of the surrounding text for better readability (as shown in Fig. R5 for quick comparison).

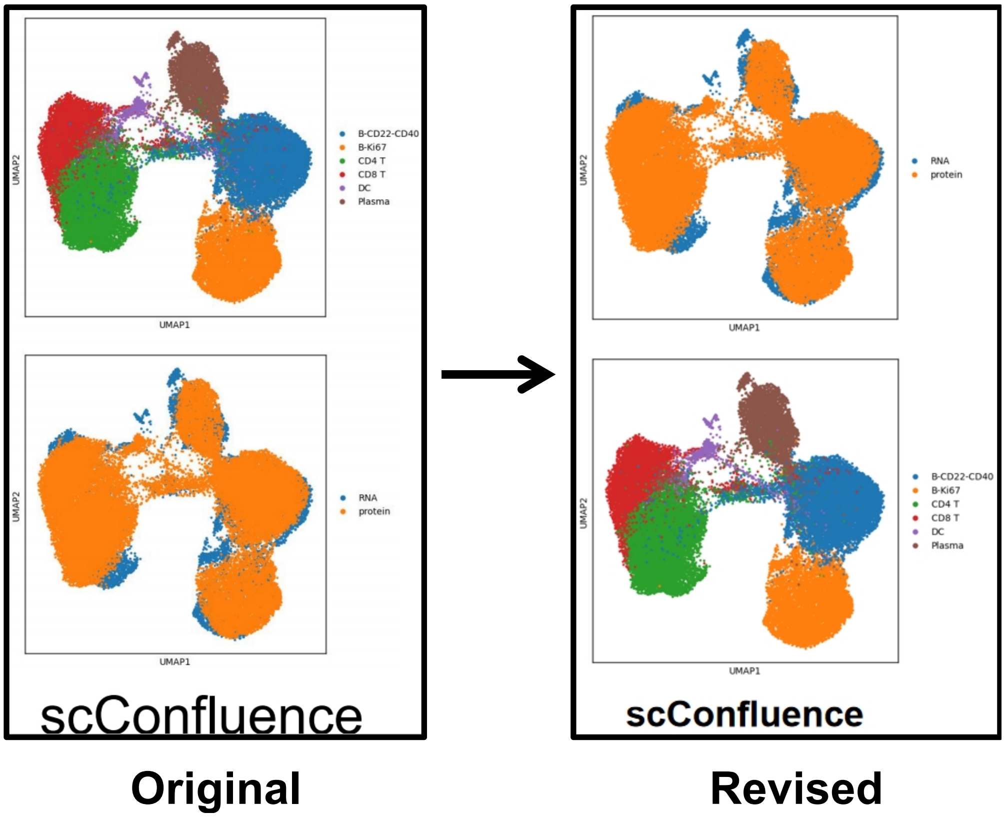

Figure R4: **Comparison of the layout logic of the UMAP subgraphs for scConfluence.** (Left) Original layout from the initial submission. (Right) Revised layout, where the subgraphs have been rearranged: the upper row is now colored by data type, and the lower row is colored by cell type.

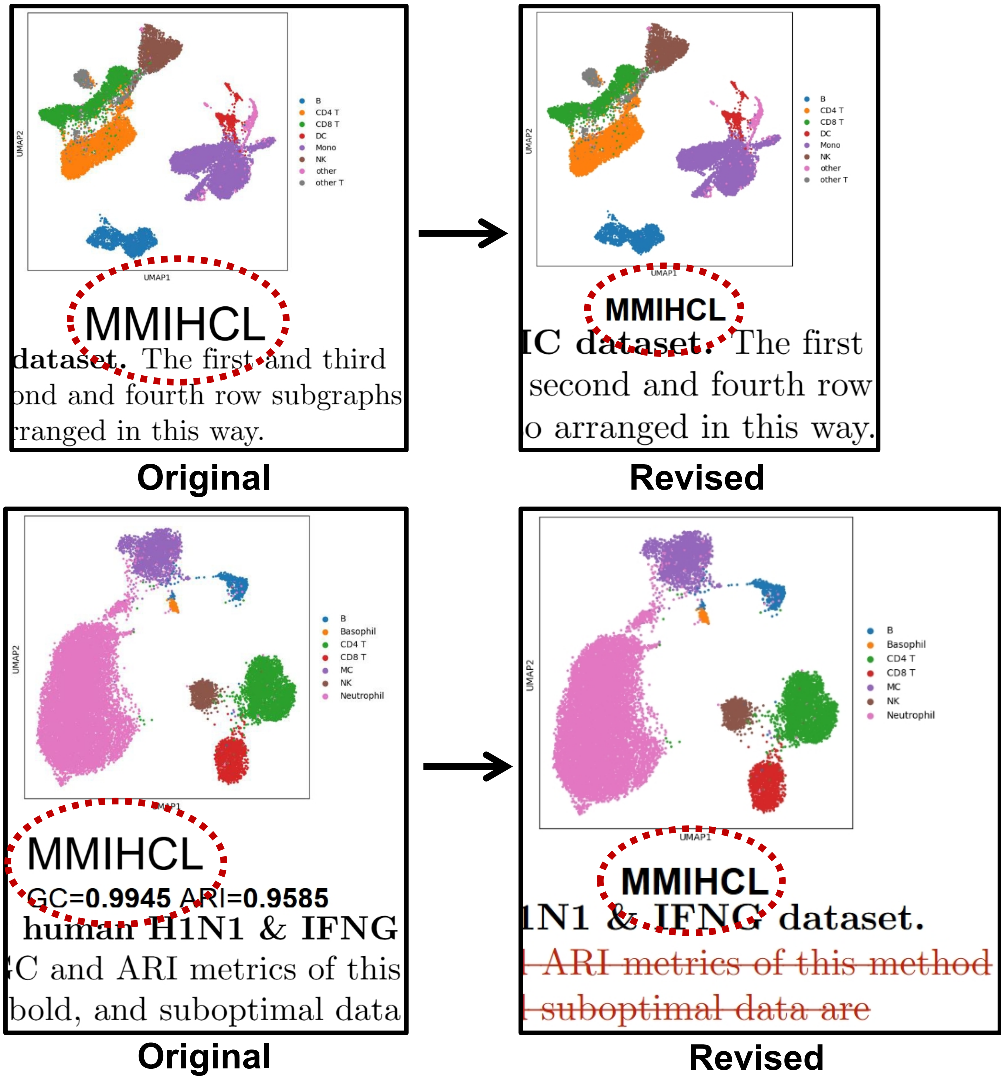

Figure R5: **Comparison of font sizes for method names.** (Left) Original versions from the initial submission. (Right) Revised versions with reduced font sizes to match the surrounding text. NOTE: The changes in the surrounding text and layout in the revised panels reflect the major content reconstruction and method updates described in previous responses, unrelated to this specific minor comment.
