# Supplementary material for: Single-cell data integration across weakly linked modalities
Source: PLoS Comput Biol. 2026 May 5;22(5):e1014231. doi: 10.1371/journal.pcbi.1014231 (PMC13160449; doi:10.1371/journal.pcbi.1014231)
Supplement: Attachment — Submitted filename: Response_to_Reviewers_auresp_2.pdf [file pcbi.1014231.s014.pdf]

Response to Editor and Reviewers for PCOMPBIOL-D-25-01825R1

Zhipeng Zhou, Yang Zhang, and Zhiming Dai

1 General Response

Dear Editor and Reviewers,

We thank the Editor and Reviewers for your continued evaluation of our manuscript (PCOMPBIOL-D-25-01825R1) and for the highly encouraging feedback on our previous revisions. We are grateful for the opportunity to submit this revised version and have carefully addressed the remaining constructive comments to further refine our work.

In this round of revision, our efforts have primarily focused on the following key updates:

- **Empirical Validation:** We have conducted comprehensive combinatorial ablation experiments and introduced a recent baseline method to rigorously demonstrate the synergistic effects among different modules and state-of-the-art performance of MMI-HCL, as suggested by Reviewer #2.
- **Presentation and Reproducibility:** We have enlarged the font size in some of the figures for better readability and provided more detailed installation instructions for our python code package, as requested by Reviewer #3.

The following sections provide our point-by-point responses to each of the comments. Please note that unless otherwise specified, all Page and Line numbers cited in this Response Letter refer to the **Revised Manuscript with Track Changes**.

Sincerely,
Zhipeng Zhou, Yang Zhang, and Zhiming Dai

2 Response to Additional Editor Comments

We thank the Editor for the encouraging comments and the opportunity to submit this revised version. We have carefully addressed the remaining concerns raised by Reviewer #2 and the formatting suggestions from Reviewer #3.

3 Response to Reviewer #1

We are grateful to the Reviewer for the positive feedback and support. We are pleased that our previous revisions addressed the reviewer's concerns, and we have further refined the

manuscript in this round to ensure the highest quality.

4 Response to Reviewer #2

Comment 1:

First, regarding my first-round Comment #4:

It is recommended to include additional ablation experiments involving the combined removal of core modules. Specifically, for the three key components (akNN, Hypergraph, and Filter), all pairwise combinations as well as the complete triple-module removal scenario should be designed as control settings. By quantifying the variation in core performance metrics under different module combinations, the following aspects should be explicitly clarified:

(1) Which module combinations exhibit significant synergistic gains;

(2) The specific contribution ratio of synergistic effects to the integration performance of weakly linked data;

(3) Whether module synergy serves as the core innovation that distinguishes MMIHCL from existing methods.

Such additional analyses would further strengthen the methodological rigor and the validity of the claimed contributions.

Response 1:

We sincerely thank the Reviewer for the continuous guidance and for pointing out the necessity of a complete modular combination analysis. We acknowledge that while the introduction of the "Baseline GCN" in the first revision provided a lower-bound reference, it was insufficient to explicitly map out the pairwise synergistic dynamics among the core components.

Strictly following your highly constructive suggestion, we have introduced additional ablation experiments encompassing all pairwise combinations of the three key modules, including w/o Filter & akNN, w/o Filter & Hypergraph CL, and w/o akNN & Hypergraph CL. Furthermore, we have explicitly designated the "Baseline GCN" as the complete triple-module removal scenario. By quantifying the performance variations across these comprehensive control settings (updated in **Table 1** and **S1 Table**), we have explicitly clarified the three critical aspects you raised in the revised **Ablation study** subsection.

(1) Which module combinations exhibit significant synergistic gains: Our research results indicate that all three different pairs of module combinations have demonstrated a profound complementary synergy, rather than a simple additive effects. Among them, the most significant performance drop occurs when both the akNN and Hypergraph CL modules are removed simultaneously. This indicates a strong interdependence during the joint embedding learning phase: the high-order structural information captured by Hypergraph CL fundamentally relies on the accurate intra-modality local geometries constructed by the akNN module. Losing either disrupts the alignment, and losing both of them simultaneously will lead to the most severe decline in matching performance.

(2) The specific contribution ratio of synergistic effects to the integration performance of weakly linked data: To address your query directly, we have quantified the specific contribution ratio in the revised manuscript in weakly linked scenarios. Our quantitative analysis reveals that the synergistic interaction between the akNN and Hypergraph CL modules yields a dominant specific contribution ratio of 86.01% (compared to Full MMIHCL, the performance difference for w/o akNN & Hypergraph CL is $\Delta S_{overall} = -0.0418$, and for Baseline GCN is $\Delta S_{overall} = -0.0486$, $|-0.0418|/|-0.0486| \approx 86.01\%$). Furthermore, other pairwise combinations also demonstrate substantial contributions (68.93% and 80.25% respectively). This explicit ratio strongly validates the indispensability of the module synergy.

(3) Whether module synergy serves as the core innovation that distinguishes MMIHCL from existing methods: We strongly agree with this perspective. The core innovation that distinguishes MMIHCL from existing methods is exactly this highly coupled, closed-loop system. Unlike conventional tools that often focus on single-perspective optimizations (e.g., solely improving GNN architectures), MMIHCL integrates intra-modality topology adaptation (akNN), cross-modality noise control (Filter), and global high-order relations (Hypergraph CL). It is this systemic synergy, rather than the mere stacking of individual components, that empowers MMIHCL to achieve state-of-the-art performance on challenging weakly linked datasets.

We believe these additional analyses and explicit clarifications significantly strengthen the methodological rigor of our study, and we are grateful to the Reviewer for prompting this deeper investigation.

Changes in Manuscript 1:

We have systematically updated the main text and supplementary materials. to incorporate the comprehensive ablation study. The specific modifications are detailed as follows:

- **Expanded the Scope of Variants:** In the **Ablation study** subsection, we expanded the original 5 MMIHCL variants to 8 control settings. We added detailed operational descriptions for the 3 newly introduced pairwise combination variants, including w/o Filter & akNN, w/o Filter & Hypergraph CL, and w/o akNN & Hypergraph CL, into the enumerated list (Page [17-18], Line [319-356]).
- **Updated Quantitative Tables:** We updated **Table 1** in the main text (Page [19]) to visually present the core performance metrics of all 8 variants under both weak and strong linkage scenarios. Concurrently, we comprehensively updated **S1 Table** in the supplementary materials to document the detailed evaluation results across all 8 datasets and 10 metrics for these newly added configurations.
- **Enhanced Textual Discussion:** We substantially revised the textual discussion (Page [18-19], Line [357-386]) analyzing the quantitative results derived from **Table 1**. Most importantly, we introduced a dedicated new paragraph to explicitly investigate the multi-module synergistic effects. This newly added paragraph directly addresses the three specific aspects highlighted by the Reviewer, including demonstrating the complementary synergistic gains, quantifying the specific contribution ratio of the synergy in the weakly linked scenario (highlighting the dominant 86.01% contribution from the akNN and Hypergraph CL combination), and establishing this highly coupled and closed-loop system as the core innovation of MMIHCL.

Comment 2:

Second, regarding my first-round Comment #5:

Although the authors have included CelLink as a baseline, the comparison set still lacks coverage of other leading technical paradigms for weak-linkage integration published after 2023. To substantiate the claim of "optimal performance", MMIHCL should be benchmarked against state-of-the-art methods from distinct methodological families.

Response 2:

We sincerely appreciate the Reviewer for this insightful and rigorous follow-up comment. We entirely agree with your assessment: evaluating MMIHCL against a single technical paradigm, even a recent SOTA like CelLink, is insufficient to conclusively establish its "optimal performance" across the diverse landscape of modern single-cell integration methods. Your push for evaluating distinct methodological families has significantly elevated the scientific nature of our benchmark assessment framework.

To directly address this critical point and substantiate our claims with comprehensive evidence, we have further expanded our experimental scope in this minor revision to include **scMRDR** (Sun et al., 2025), a highly scalable and flexible generative framework recently developed for unpaired multi-omics integration. The inclusion of **scMRDR** squarely addresses your requirement for methodological diversity. While our previously added baseline, CelLink, represents the cutting-edge mathematical paradigm based on an iterative Unbalanced Optimal Transport (UOT) framework, the newly introduced **scMRDR** embodies the state-of-the-art in the Generative Deep Learning (GDL) paradigm via its regularized β-VAE architecture. By evaluating MMIHCL against both UOT-based and GDL-based methods, our comparative set now comprehensively covers the leading technical paradigms published after 2023.

Based on this expanded framework, we have updated all quantitative and qualitative evaluations. Although the newly added computational cost analysis highlights **scMRDR**'s unparalleled spatio-temporal efficiency and highly competitive alignment in strongly linked datasets, our multi-dimensional assessments confirm that MMIHCL consistently maintains the highest performance ceiling. Particularly in challenging weak-linkage scenarios, where pure generative alignment struggles with severe feature sparsity, MMIHCL's adaptive neighborhood and hypergraph contrastive learning mechanisms uniquely ensure superior biological fidelity and accurate cell-type matching.

Consequently, by explicitly demonstrating MMIHCL's sustained superiority against the most recent state-of-the-art models from entirely distinct methodological families, the claim of "optimal performance" is now substantiated with more solid, rigorous, and diverse comparative evidence. We sincerely thank the Reviewer for guiding us to achieve this level of methodological completeness.

Changes in Manuscript 2:

In direct response to the Reviewer's highly constructive feedback regarding the necessity of covering "other leading technical paradigms" published after 2023, we have extensively revised the manuscript to incorporate **scMRDR** as a core baseline. Building upon our previous integration of CelLink, this addition represents a comprehensive and systematic update spanning from the theoretical background in the **Introduction** section to the rigor-

ous, multi-dimensional benchmarking in the **Results** section.

Firstly, we have systematically updated the textual and theoretical framework of the manuscript to explicitly integrate **scMRDR**'s distinct technical paradigm. In the **Introduction** section, we added the description (Page [3], Line [74-76]): "Furthermore, scMRDR further enhances the scalability and flexibility of unpaired integration across diverse modalities by learning regularized disentangled representations." Correspondingly, we updated the baseline declarations in the **Results** section to formally incorporate it into our comparative framework: "We used seven methods published for multimodal integration as baselines, including... CelLink, and scMRDR." (Page [6], Line [127-131]). Furthermore, to guarantee experimental reproducibility, the detailed implementation protocols for **scMRDR** —— including its data preprocessing, hyperparameter configurations, and cross-modality prediction workflow —— have been documented in the **Methods for benchmarking** section in **S1 File** (Page [3], Line [84-99]).

Secondly, to empirically validate MMIHCL's sustained superiority against this distinct generative paradigm, we have meticulously updated our rigorous benchmarking framework to evaluate **scMRDR** across multiple quantitative and qualitative dimensions.

- **Comprehensive Performance Benchmarking:** We systematically incorporated **scMRDR** into our multi-dimensional quantitative evaluations across both weakly and strongly linked datasets. Our updated results provide concrete statistical evidence of MMIHCL's sustained optimal position in challenging scenarios. As highlighted in the revised text regarding weak linkage integration (Page [6-7], Line [147-150]): "Notably, while the recently introduced scMRDR achieves a moderate overall score, it still lags behind MMIHCL by a notable margin ($\Delta = -0.1477$)..."
- **Spatio-temporal Efficiency Analysis:** To provide a more holistic and unbiased evaluation, we introduced a detailed benchmarking of computational cost and scalability. In this section, we explicitly recognized the structural advantages of **scMRDR**'s generative paradigm. As stated on Page [23], Line [464-466]: "Notably, the recently proposed scMRDR exhibited an unparalleled spatio-temporal efficiency across these benchmarks, achieving an even lower average running time (6.8312 minutes) and an extremely minimal memory usage (0.0475 GB)."
- **Expanded Visual Evidence:** We have thoroughly updated all corresponding figures to visually benchmark **scMRDR**. Specifically, we integrated scMRDR into the performance, downstream, and cost analysis in **Fig. 2, 3, 4, 5, 6, 9** (Page [7, 9, 10, 12, 13, 23]), and **S1-7 Fig**. Through these comprehensive visual updates, the robust superiority of MMIHCL is demonstrated in an intuitive and vivid manner.

5 Response to Reviewer #3

Comment 1:

To improve the clarity and readability of the figures, I suggest the authors enlarge the font size, as some of the text appears too small in its current form.

Response 1:

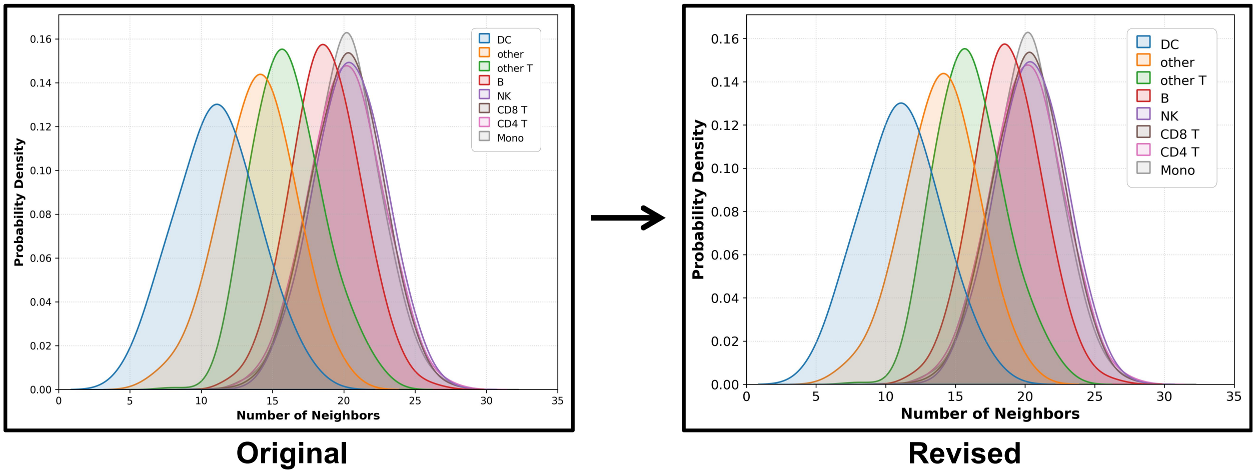

Figure R1: **Comparison of the original and revised font sizes in a representative statistical graph.** In this representative quantitative statistical graph (Fig. 8c in the main text), key textual elements including axis labels, tick labels, and legends have been systematically enlarged.

We sincerely thank the Reviewer for this helpful suggestion. We entirely agree that ensuring the clarity and readability of the figures is crucial for presenting our quantitative evaluations effectively.

In accordance with your advice, we have systematically enlarged the vast majority of fonts across all statistical graphs involving quantitative evaluations in both the main text and the supplementary materials. Specifically, we have up-scaled key elements such as axis labels, tick labels, and legends, thereby significantly enhancing the overall visual clarity and ensuring that the fine details of our quantitative results are effortlessly readable.

Changes in Manuscript 1:

We have systematically enlarged the vast majority of text elements across all quantitative statistical graphs. Taking **Fig. 8c** from the main text (Page [20]) as a representative example, a visual comparison demonstrating the up-scaled axis labels (from 12 pt to 15 pt), tick labels, and legends (from 10 pt to 13 pt) is provided below (see Fig. R1).

Due to space constraints, we are unable to display the "Before & After" comparisons for every revised figure in this response letter. We kindly refer the Reviewer to the revised manuscript and supplementary materials to review all the comprehensively updated figures.

Comment 2:

For the installation instruction of the package, please also provide a clear command to install it.

Response 2:

We sincerely thank the Reviewer for this valuable suggestion, which greatly helps readers configure the environment and reproduce our work more effortlessly. Upon reviewing our original `README.md` file, we realized that the previous installation instructions had the following limitations:

- **Suboptimal Prioritization:** The highly efficient installation files (`requirements.txt` and `environment.yml`) were only mentioned as an afterthought at the end of the guide. Conversely, a tedious line-by-line manual installation process was presented as the primary method.
- **Incomplete Workflow:** The instructions lacked the essential initial step to acquire the code repository (i.e., the `git clone` command). Additionally, the absence of automation flags (such as `-y` in the conda command) prevented a seamless, one-click copy-and-paste setup experience.

Therefore, we fully agree with the Reviewer that it is essential to provide a clear command to install MMIHCL, as this will minimize potential setup errors and ensure a seamless, out-of-the-box experience for the research community.

Changes in Manuscript 2:

We have completely updated the `README.md` file in our official GitHub repository (https://github.com/SundayChou/MMIHCL). Readers can visit this link directly to view the latest, reader-friendly installation instructions.

As illustrated in Fig. R2, the revised installation guide overcomes the limitations of the previous version through the following structural improvements:

- **Prioritizing One-Click Installation:** We now prominently feature a "Quick Installation" section at the very beginning. It provides the clear, single command requested by the Reviewer (e.g., `pip install -r requirements.txt`), replacing the tedious manual steps as the primary recommendation.
- **Completing the Setup Workflow:** We have added the essential `git clone` and directory navigation (`cd`) commands, along with the `-y` flag in the conda environment creation step. This ensures a truly seamless, unbroken copy-and-paste execution from start to finish.
- **Structuring for Flexibility:** The original line-by-line instructions have been preserved but logically reorganized into an alternative "Step-by-step Manual Installation" section, catering exclusively to users who require specific PyTorch and CUDA configurations for their hardware.

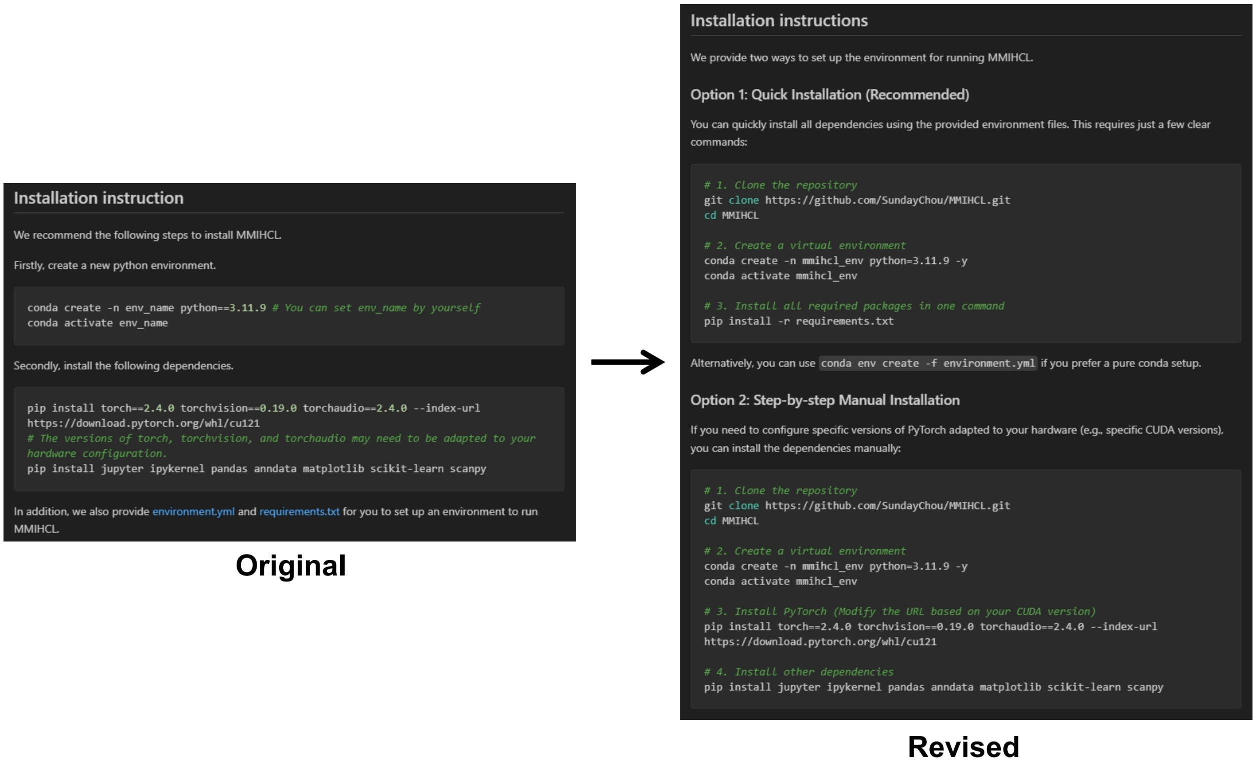

Figure R2: **Comparison of the original and revised installation instructions in the GitHub repository.** The revised version introduces a structured "Quick Installation" option that provides a clear, one-line command (`pip install -r requirements.txt`) for environment setup. Additionally, it reorganizes the original step-by-step guide into a "Manual Installation" section to accommodate users requiring specific hardware adaptations.
